# Germline mutational spectrum in Armenian breast cancer patients suspected of hereditary breast and ovarian cancer

**DOI:** 10.1038/s41439-021-00140-2

**Published:** 2021-02-09

**Authors:** Mike M. Moradian, Davit T. Babikyan, Sione Markarian, Jonny G. Petrosyan, Nare Avanesian, Tereza Arutunyan, Tamara F. Sarkisian

**Affiliations:** 1 Department of Medical Genetics, Yerevan State Medical University, Yerevan, Armenia; 2 Department of Molecular Genetics, Morava Scientific & Technology Services, Glendale, CA USA; 3 Department of Molecular Genetics, Center of Medical Genetics and Primary Health Care, Yerevan, Armenia

**Keywords:** Cancer genetics, Cancer genetics

## Abstract

Hereditary breast and ovarian cancer (HBOC) can be identified by genetic testing of cancer-causing genes. In this study, we identified a spectrum of genetic variations among 76 individuals of Armenian descent either with a family history of cancer or breast cancer before the age of 40. We screened 76 suspected HBOC patients and family members as well as four healthy controls using a targeted and hereditary comprehensive cancer panel (127 genes). We found 26 pathogenic (path) and 6 likely pathogenic (LPath)variants in 6 genes in 44 patients (58%); these variants were found in *BRCA1* (17), *BRCA2* (19), *CHEK2* (4), *PALB2* (2), and *NBN* (1). A few different variants were found in unrelated individuals; most notably, variant p.Trp1815Ter in the *BRCA1* gene occurred in four unrelated patients. We did not find any known significant variants in five patients. Comprehensive cancer panel testing revealed pathogenic variants in cancer genes other than *BRCA1* and *BRCA2*, suggesting that testing only *BRCA1* and *BRCA2* would have missed 8 out of 44 suspected HBOC patients (18%). These data also confirm that a comprehensive cancer panel testing approach could be an appropriate way to identify most of the variants associated with hereditary breast cancer.

## Breast cancer: The spectrum of mutations in Armenian patients

Genetic variants associated with hereditary breast cancer have been identified by a comprehensive analysis of Armenian patients, highlighting the need for testing panels to be tailored to the population being screened. A team led by Mike Moradian and Davit Babikyan of Yerevan State University Medical School, screened samples from 76 breast cancer patients of Armenian descent. Using a panel including 127 genes, they identified 32 pathogenic variants in 44 patients, mostly in *BRCA1* or *BRCA2*. However, testing only *BRCA1* and *BRCA2* would have missed 8 of these 44 patients. The analysis also found variants of unknown significance in 32 patients which might be involved in hereditary breast cancer. Based on this analysis, the researchers argue that it is important to screen with comprehensive gene panels to increase the chance of detecting variants associated with cancer.

## Introduction

Hereditary breast and ovarian cancer (HBOC) and their causative variants have become one of the most studied hereditary cancers. Although the *BRCA1* (NM_007294.3) and *BRCA2* (NM_000059.3) gene variants were the primary focus of such studies in the past, hereditary cancer panel testing has recently replaced the approach involving only the *BRCA1* and *BRCA2* genes. In fact, it has been shown that close to 10% of hereditary breast cancer can be caused by variants in genes such as *CHEK2* (NM_007194.3), *PALB2* (NM_024675.3), *ATM* (NM_000051.3), and *MUTYH* (NM_001128425.1)^1^. There are hundreds of genes that could be involved in tumorigenesis and cancer, yet not all are involved in every cancer. Thus, there are specific hereditary cancer panels for breast, ovarian, colorectal, endometrial, prostate, and other cancers. These panels contain some of the most prevalently mutated genes in each specific cancer, yet there could be genes that may cause hereditary cancer in a specific population that may not be included in general cancer gene panels. Therefore, ethnic-specific population studies could be important in the identification and inclusion of genes that may cause hereditary cancers in a specific population.

Additionally, there are several cancer-causing variants that could be more prevalent in an ethnic or specific population. The most famous of these variants are the three *BRCA1* and *BRCA2* variants (i.e., 5382insC and 185delAG in *BRCA1* and 6174delT in *BRCA2*) that are prevalent in the Ashkenazi Jewish population^2^. Ethnic-specific studies have shown that there may be variants classified as founder mutations, as well as unique variants in suspected HBOC patients. In fact, the number of founder mutations compared to unique variants is quite small, indicating that some populations will not benefit from targeted gene testing. Recently, gene panels have been shown to be the better approach, expanding HBOC variant discovery to several genes from different pathways (Breast Cancer Information Core, BIC). This approach has resulted in the identification of many pathogenic (Path) and likely pathogenic (LPath) variants, and variants of unknown significance (VUSs) from genes other than *BRCA1* and *BRCA2*. Path and LPath variants are important in drug discovery and treatment options, while VUSs could be significant in the initiation of more studies to identify their cancer-causing roles. Some deleterious variants could introduce premature termination codons through frameshift deletions or insertions, nonsense or splice junction mutations, or large deletions or duplications. Some splice site mutations and large rearrangements do not change the reading frame but result in a loss or gain of one to several exons, which could potentially have an impact on gene function. Deleterious missense mutations are typically confined within specific residues of functional motifs. However, the risk contribution of numerous other sequence variants remains unclear. Some VUSs include missense changes and small in-frame deletions and insertions that mostly lead to one amino acid change without a frameshift as well as alterations in noncoding intervening sequences or in untranslated exonic regions^3^.

There have been two small-scale studies on breast cancer genetics in the Armenian population. One study attempted to find known *BRCA1* and *BRCA2* founder mutations in 46 suspected HBOC patients, but no founder mutations were found^4^. Another study attempted to identify *BRCA1* and *BRCA2* mutations in six suspected HBOC patients of Armenian descent using targeted panel testing. No pathogenic variants were found in these patients^5^.

In this study, we analyzed the mutation spectrum of 76 patients of Armenian descent with suspicion of hereditary breast cancer (family history of cancer) or breast cancer under the age of 40, selected according to the National Comprehensive Cancer Network (NCCN) guidelines, using a comprehensive 127-gene hereditary cancer panel. The purpose of this study was not only to identify the mutational spectrum of HBOC in the Armenian population but also to identify the genes that could have breast cancer-causing variants in the Armenian population. We intended to identify these genes to provide insights for an Armenian-specific HBOC-testing gene panel and a comparison with genes in panels for other ethnic groups. We also anticipated finding novel variants that could potentially cause breast cancer due to potential different distributions of disease-causing variants in the Armenian population. This is the first large-scale study of breast cancer-causing variants in the Armenian population.

## Materials and methods

### Patient selection and BRCAPRO score

We tested 76 suspected HBOC patients or family members and 4 healthy controls using a targeted test (24 patients) and a hereditary comprehensive cancer panel (127 genes). Overall, 34 patients were <40 years of age (22 with a family history of cancer, 63%), 16 patients were between 40 and 50 years old (10 with a family history of cancer, 63%), and 26 patients were >50 years of age (25 with a family history of cancer, 96%). All patients were selected according to the criteria provided in the NCCN Clinical Practice Guidelines in Oncology (Version 2.2019-July 30.2018). Only patients with a confirmed histopathologic diagnosis of invasive breast cancer (BC) were included in the study. Following genetic counseling, the probability for each female patient to be a mutation carrier in one or both of the *BRCA* genes was estimated using the BRCAPRO model of the University of Texas Southwestern Medical Center at Dallas CancerGene software, version 5.1 (http://www4.utsouthwestern.edu/breasthealth/cagene/). The BRCAPRO risk is derived through the Bayesian probability model and takes into account the first and second degree relatives of a patient, age at the time of diagnosis of BC and/or ovarian cancer (OC), and ages of unaffected family members^6^. The male patients with a diagnosis of BC were not subject to BRCAPRO risk calculation given that the model values for male BC patients were significantly higher than those for female BC patients. Patients were all ethnically Armenian. This study was approved by the Institutional Review Board of the Center of Medical Genetics and Primary Health Care. Informed consent was obtained from all human subjects participating in this research.

### Gene panel sequencing and bioinformatics

Variant analysis was performed using an advanced bioinformatics pipeline and manual curation.

A 127-gene comprehensive cancer panel *(AIP, ALK, APC, ATM, ATR, AXIN2, BAP1, BARD1, BLM, BMPR1A, BRCA1, BRCA1, BRIP1, BUB1B, CASR, CDC73, CDH1, CDK4, CDKN1B, CDKN1C, CDKN2A, CEBPA, CHEK2, CTC1, CTNNA1, CYLD, DDB2, DICER1, DIS3L2, DKC1, EGLN1, EPCAM, ERCC1, ERCC2, ERCC3, ERCC4, ERCC5, EXT1, EXT2, EZH2, FAN1, FANCA, FANCB, FANCC, FANCD2, FANCE, FANCF, FANCG, FANCI, FANCL, FANCM, FH, FLCN, GALNT12, GATA2, GPC3, GREM1, HOXB13, HRAS, KIF1B, KIT, LZTR1, MAX, MC1R, MEN1, MET, MITF, MLH1, MLH3, MRE11, MSH2, MSH6, MUTYH, NBN, NF1, NF2, NHP2, NOP10, NTHL1, PALB2, PDGFRA, PHOX2B, PMS2, POLD1, POLE, POLH, POT1, PRKAR1A, PRSS1, PTCH1, PTCH2, PTEN, RAD50, RAD51C, RAD51D, RB1, RECQL4, RET, RUNX1, SDHA, SDHAF2, SDHB, SDHC, SDHD, SLC45A2, SLX4, SMAD4, SMARCA4, SMARCB1, SMARCE1, STK11, SUFU, TERC, TERT, TINF2, TMEM127, TP53, TSC1, TSC2, TYR, VHL, WRAP53, WRN, WT1, XPA, XPC*, and *XRCC2)* offered by Fulgent Diagnostics (Temple City, CA) was used to sequence 76 patients and 4 healthy controls. Next-generation sequencing (NGS) was performed on a HiSeq instrument from Illumina, La Jolla, CA. The average depth of sequencing depth/mean coverage was between 1100x and 1300x. The assay mainly covers the exonic coding regions 10–20 bp into the flanking introns. The promoter region of the *PTEN* gene was sequenced, and the promoter regions of the *BRCA1* and *BRCA2* genes were not included in the panel. *PMS2* pseudogene detection was performed by Fulgent’s proprietary “Misalignment Tool”, which analyzed both reads from the real gene and the pseudogene. Some patients were also confirmed by long-range PCR for mutations in exon 13 to exon 15 of the *PMS2* gene. After sequencing, the Fulgent bioinformatics pipeline was used to obtain a list of variants with data from standards and guidelines for the interpretation of sequence variants recommended by the American College of Medical Genetics and Genomics (ACMG)^7,8^. Each variant for every sample was analyzed in detail before any classification was made.

### FATHMM, SIFT, PolyPhen-2, and CADD

#### FATHMM

The program Functional Analysis Through Hidden Markov Models (FATHMM) predicts whether single nucleotide variants (SNVs) in the human genome are likely to be functional or nonfunctional in inherited diseases. FATHMM uses distinct models for coding and noncoding regions to improve overall accuracy. The coding predictor is based on six groups of features representing sequence conservation, nucleotide sequence characteristics, genomic features (codons, splice sites, etc.), amino acid features and expression levels in different tissues. We used a threshold of 0, which generated a sensitivity of 0.94^9^.

#### Sorting intolerant from tolerant (SIFT)

The program sorting intolerant from tolerant (SIFT) predicts whether an amino acid substitution is likely to affect protein function based on sequence homology and the physicochemical similarity between the alternate amino acids^10^. The data we provide for each amino acid substitution are a score and a qualitative prediction (either “tolerated” or “deleterious”). The score is the normalized probability that the amino acid change is tolerated, so scores closer to zero are more likely to be deleterious. The qualitative prediction is derived from this score such that substitutions with a score <0.05 are called “deleterious”, and all others are called “tolerated”. We used the criteria of <0.05 as damaging and between 0.05–0.07 as probably damaging.

#### PolyPhen-2

The program PolyPhen-2 predicts the effects of an amino acid substitution on the structure and function of a protein using sequence homology, Pfam annotations, 3D structures from PDB where available, and a number of other databases and tools (including DSSP and ncoils). The PolyPhen score represents the probability that a substitution is damaging, so values closer to one are more confidently predicted to be deleterious^11^. The qualitative prediction is based on the false- positive rate of the classifier model used to make the predictions. We used the following criteria: scores of 0.446–0.908 as probably damaging and scores of 0.908 or more as damaging.

#### Combined annotation-dependent depletion (CADD)

The combined annotation-dependent depletion (CADD) tool scores the predicted deleteriousness of single nucleotide variants and insertion/deletion variants in the human genome by integrating multiple annotations, including conservation and functional information, into one metric. Phred-style CADD raw scores are displayed, and variants with higher scores are more likely to be deleterious. CADD provides genome wide scores^12^. CADD provides a ranking rather than a prediction or default cutoff, with higher scores more likely to be damaging. We used the following criteria: scores of 10–20 as probably damaging and scores of >20 as damaging.

### Variants

All variants reported in this study were described according to current HGVS mutation nomenclature guidelines and were verified using Variant Validator^13^. All variants identified in this study were submitted to the ClinVar database.

## Results

### Pathogenic or likely pathogenic variants

We found 44 patients with pathogenic or likely pathogenic variants (32 unique variants) in 76 tested patients or family members (58%). The number of patients with *BRCA1* and *BRCA2* variants was 36, ~82% of this group. These variants were closely split, with 19 patients with *BRCA2* (14 unique) and 17 patients with *BRCA1* (10 unique) variants. The remaining variants (i.e., 9) were identified in the following genes: *BRIP1* (1), *CHEK2* (4), *NBN* (1), and *PALB2* (2). The majority of the *BRCA1* variants were either frameshift or nonsense mutations with 6 frameshift, 1 nonsense, 1 missense, and 2 intronic mutations. The *BRCA2* gene had a slightly different variant comprising 8 frameshift, 4 nonsense, and 2 missense mutations. Variants in the rest of the genes were a mix of frameshift, nonsense, and missense mutations (Table 1). Variant p.Trp1815Ter in the *BRCA1* gene was seen in four unrelated patients, all with a family history of cancer, indicating a possible founder mutation in the Armenian population. Variant p.Ile1159Metfs in *BRCA1* was identified in the same family members from two different families, and p.Leu1669Ter in *BRCA2* was identified in two sisters and one unrelated individual. Additionally, each one of the variants p.Cys1146Leufs in *BRCA1* and p.Ala938Profs in *BRCA2* were present in two unrelated patients. These data suggest that these variants may also be founder mutations. *BRCA1* gene variants were found in three different exons: exon 5 (1), exon 11 (6), and exon 23 (1). The *BRCA2* gene variants were found in seven different exons: exon 7 (1), exon 10 (2), exon 11 (7), exon 16 (1), exon 22 (1), exon 23 (1), and exon 25 (1). Two of the pathogenic variants in the *BRCA1* gene were splice acceptor site variants (NM_007294.3: c.302-1G>A) and (NM_007294.3: c.4358-2A>G). These splice site mutations could result in a truncated or altered protein, potentially interfering with its function in DNA repair. Other mutations have been reported in the splice site at positions *BRCA1* c.302-1 or c.302-2 (source, ClinVar).

**Table 1:** Patients with only pathogenic and likely pathogenic variants.

Patient ID	Gender	Exon	Gene name	Variant (cDNA)	Variant (protein)	AOD	Cancer type	Family history of cancer	BRCAPRO score (%)	Variant classification
P-01	F	5	*BRCA1*	c.211A>G	p.Arg71Gly	36	UBC	BRC in 1st degree relative	34.20%	PATH
P-02	F	–	*BRCA1*	c.302-1G>A	-	32	UBC	No family history reported	6.20%	PATH
P-03	F	11	*BRCA1*	c.798_799del	p.Ser267Lysfs	56	UBC	BRC in 1st degree relative	8.00%	PATH
P-04	F	11	*BRCA1*	c.1504_1508delTTAAA	p.Leu502Alafs	40	BBC	No family history reported	5.90%	PATH
P-05	F	11	*BRCA1*	c.2649_2650insGGCA	p.Thr884Glyfs	36	UBC	Moth LUC (42); Mat G.Moth OC (57)	24.00%	PATH
P-06	F	11	*BRCA1*	c.3436_3439delTGTT	p.Cys1146Leufs	32	UBC	BRC in 1st degree relative	41.50%	PATH
P-07	F	11	*BRCA1*	c.3436_3439delTGTT	p.Cys1146Leufs	45	UBC	BRC in 1st degree relative	12.80%	PATH
P-08^a^	F	11	*BRCA1*	c.3477_3480delAAAG	p.Ile1159Metfs	31	UBC	Sis BRC	50.40%	PATH
P-09^a^	F	11	*BRCA1*	c.3477_3480delAAAG	p.Ile1159Metfs	32	UBC	Sis BRC	50.40%	PATH
P-10^a^	F	11	*BRCA1*	c.3477_3480delAAAG	p.Ile1159Metfs	54	UBC	Bro BRC	3.50%	PATH
P-11^a^	M	11	*BRCA1*	c.3477_3480delAAAG	p.Ile1159Metfs	57	UBC	Sis BRC	N/A	PATH
P-12	F	11	*BRCA1*	c.3485delA	p.Asp1162Valfs	51	UBC	Sis OC (42); Daughter OC (32)	85.30%	PATH
P-13^b^	F	–	*BRCA1*	c.4358-2A>G	-	40	OC&BC	Moth OC (50); Pat G.Moth LUC; Sis OC	99.70%	LPATH
P-14	F	23	*BRCA1*	c.5444G>A	p.Trp1815Ter	26	UBC	Moth BRC (32); Mat G.Moth BRC (34); Mat G.G.Moth BRC (45); Moth Mat Aunts BRC (36&37)	92.30%	PATH
P-15	F	23	*BRCA1*	c.5444G>A	p.Trp1815Ter	29	UBC	Pat G.Moth BRC (60); Pat Aunt: BRC (46)	33.30%	PATH
P-16	F	23	*BRCA1*	c.5444G>A	p.Trp1815Ter	38	UBC	Sis BRC & UterC; Bro StomC	36.40%	PATH
P-17	M	23	*BRCA1*	c.5444G>A	p.Trp1815Ter	65	UBC	Moth BRC (50); Pat Aunt: BRC (55)	N/A	PATH
P-18	F	7	*BRCA2*	c.574dupA	p.Met192Asnfs	36	BBC	No family history reported	15.10%	PATH
P-19	F	10	*BRCA2*	c.1414C>T	p.Gln472Ter	53	UBC	Moth BRC (59); Sis BRC (42); Sist BRC (50); Mat Aunt: BRC (68 & 72); Mat G.fath ProsC (65); Mat G.Moth OC (43)	81.30%	PATH
P-20	F	10	*BRCA2*	c.1528G>T	p.Glu510Ter	55	UBC	BRC in 1st degree relative	10.10%	PATH
P-21	F	11	*BRCA2*	c.2095C>T	p.Gln699Ter	30	UBC	Pat G.Moth UterC	6.90%	PATH
P-22^a^,^b^	F	11	*BRCA2*	c.2623G>C	p.Val875Leu	51	UBC	Sis BRC (44); Mat G.Moth BRC (87)	9.20%	LPATH
P-23^a^,^b^	F	11	*BRCA2*	c.2623G>C	p.Val875Leu	44	UBC	Sis BRC (51); Mat G.Moth BRC (87)	9.70%	LPATH
P-24	F	11	*BRCA2*	c.2808_2811delACAA	p.Ala938Profs	29	UBC	BRC in 1st degree relative	71.60%	PATH
P-25	F	11	*BRCA2*	c.2808_2811delACAA	p.Ala938Profs	38	UBC	Moth BRC (39); Mat Aunt: BRC (40)	75.00%	PATH
P-26	F	11	*BRCA2*	c.4037_4038delCT	p.Thr1346Serfs	53	UBC	BRC in 1st degree relative	11.10%	PATH
P-27	F	11	*BRCA2*	c.4548_4549delCA	p.Lys1517Argfs	62	UBC	BRC in 1st degree relative	7.00%	PATH
P-28	F	11	*BRCA2*	c.5006T>G	p.Leu1669Ter	81	UBC	BRC in 1st degree relative	1.60%	PATH
P-29^a^	F	11	*BRCA2*	c.5006T>G	p.Leu1669Ter	26	UBC	Sis BRC (56)	20.60%	PATH
P-30^a^	F	11	*BRCA2*	c.5006T>G	p.Leu1669Ter	56	UBC	Sis BRC (26)	17.30%	PATH
P-31	F	11	*BRCA2*	c.6302delA	p.Asn2101Metfs	31	UBC	Mat Aunt: BRC (36); Sec Cous BRC (43)	29.00%	PATH
P-32	F	16	*BRCA2*	c.7689delC	p.His2563Glnfs	38	UBC	BRC in 1st degree relative	28.70%	PATH
P-33	M	16	*BRCA2*	c.7689delC	p.His2563Glnfs	68	UBC	BRC in 1st degree relative	N/A	PATH
P-34^b^	F	22	*BRCA2*	c.8851G>T	p.Ala2951Ser	49	UBC	Sis BRC (38); Pat Aunt BRC (70); Mat G.Moth BRC	34.20%	LPATH
P-35	F	23	*BRCA2*	c.9097dupA	p.Thr3033Asnfs	48	UBC	BRC in 1st degree relative	10.10%	PATH
P-36	F	25	*BRCA2*	c.9253delA	p.Thr3085Glnfs	41	UBC	Moth BRC (46)	17.70%	PATH
P-37	F	7	*BRIP1*	c.917dup	p.Asn306Lysfs	59	UBC	BRC in 1st degree relative	7.00%	PATH
P-38	F	3	*CHEK2*	c.409C>T	p.Arg137Ter	54	UBC	BRC in 1st degree relative	10.30%	PATH
P-39^b^	F	3	*CHEK2*	c.422A>C	p.Lys141Thr	45	UBC	Moth BRC (57); Sis BRC (34); Mat Aunt UterC (52)	42.00%	LPATH
P-40	F	4	*CHEK2*	c.470T>C	p.Ile157Thr	46	UBC	BRC in 1st degree relative	18.20%	LPATH
P-41	F	4	*CHEK2*	c.499G>A	p.Gly167Arg	54	UBC	BRC in 1st degree relative	10.30%	LPATH
P-42	F	11	*NBN*	c.1502G>A	p.Trp501Ter	51	UBC	BRC in 1st degree relative	12.90%	PATH
P-43	F	4	*PALB2*	c.932_933insC	p.Lys311Asnfs	68	UBC	Bro ProsC (62)	3.30%	PATH
P-44	F	12	*PALB2*	c.3299_3306dup	p.Val1103Leufs	51	UBC	BRC in 1st degree relative	12.90%	PATH

Complete patient and variant information and family history for identified pathogenic and likely pathogenic mutations.

*P* patient, *PATH* pathogenic, *LPATH* likely pathogenic, *AOD* age of diagnosis, *BRC* breast cancer, *OC* ovarian cancer, *LUC* lung cancer, *UterC* uterine cancer, *StomC* stomach cancer, *ProsC* prostate cancer, *Moth* mother, *Fath* father, *Sis* sister, *Bro* brother, *Mat* maternal, *Pat* paternal, *G* grand, *UBC* unilateral breast cancer, *BBC* bilateral breast cancer, *N/A* not applicable.

^a^Sibling pairs.

^b^Patient also had a VUS variant.

We found four patients in this patient cohort that did not report any family history of cancer yet had pathogenic or likely pathogenic variants identified. Three of them had bilateral breast cancer, and one had unilateral breast cancer before the age of 35. Additionally, to the best of our knowledge, one of the identified variants, p.Val875Leu, in *BRCA2* has not been reported in any patient suspected of HBOC. A detailed explanation of every pathogenic and likely pathogenic variant annotation is presented in the supplementary material.

### Variants of unknown significance (VUSs)

We found 37 variants of unknown significance (VUSs) in 32 patients, and based on our bioinformatics pipeline designation, these variants had indications of being significant but are not well studied or reported (Table 2). We reported a 45% VUS rate, which is higher than the industry-reported 30–40% VUS rate. A detailed explanation of every VUS annotation is presented in the supplementary material. We did not analyze the following VUSs: (*MUTYH* p.Leu420Met; CHEK2 p.Asp438Tyr; *SLX4* p.Cys1805Arg; and *MLH1* p.His318Gln) in patients who also carried likely pathogenic variants; these variants are only reported in the Supplemental Table.

**Table 2:** Patients with variants of unknown significance (VUS).

Variant	Gender	Exon	Gene name	Variant (cDNA)	Variant (protein)	AOD	Cancer type	Family history of cancer	BRCAPRO score (%)
VUS-01^b^ P-45	F	18	*ATM*	c.3371A>T	p.Tyr1124Phe	26	UBC	Pat 2 cousins BRC; Moth’s Pat Uncle ProstC	8.40%
VUS-02^b^ P-45	F	3	*XRCC2*	c.268C>T	p.Leu90Phe	26	UBC	Pat 2 cousins BRC; Moth’s Pat Uncle ProstC	8.40%
VUS-03 P-46	F	15	*SLX4*	c.5413T>C	p.Cys1805Arg	26	UBC	No family history reported	8.50%
VUS-04^b^ P-47	F	11	*RECQL4*	c.2704C>T	p.Arg902Trp	27	UBC	No family history reported	8.10%
VUS-05^b^ P-47	F	10	*POLE*	c.5653G>A	p.Ala1885Thr	27	UBC	No family history reported	8.10%
VUS-06^b^ P-48	F	8	*AXIN2*	c.2083G>T	p.Ala695Ser	28	UBC	No family history reported	7.70%
VUS-07^b^ P-48	F	2	*TSHR*	c.202C>T	p.Pro68Ser	28	UBC	No family history reported	7.70%
VUS-08^b^ P-49	F	11	*SLX4*	c.2320G>T	p.Ala774Ser	28	UBC	Moth BRC (44); Mat G.Moth EsophC (75); G.Fath GastC (62); Mat Aunt GastC (50); Pat Aunt ColorecC (60)	21.40%
VUS-09^b^ P-49	F	10	*WRAP53*	c.1564delG	p.Ala522Argfs	28	UBC	Moth BRC (44); Mat G.Moth EsophC (75); G.Fath GastC (62); Mat Aunt GastC (50); Pat Aunt ColorecC (60)	21.40%
VUS-10^b^ P-50	F	21	*BRCA2*	c.8699A>T	p.Asp2900Val	29	UBC	Moth Melanoma	5.70%
VUS-11^b^ P-50	F	2	*SLX4*	c.421G>T	p.Gly141Trp	29	UBC	Moth Melanoma	5.70%
VUS-12 P-51	F	-	*BRCA1*	c.-86C>T	-	30	UBC	No family history reported	6.90%
VUS-13 P-52	F	21	*BRCA1*	c.5360G>A	p.Cys1787Tyr	31	UBC	No family history reported	5.90%
VUS-14 P-53	F	6	*WRAP53*	c.838G>A	p.Ala280Thr	31	UBC	No family history reported	6.50%
VUS-15 P-54	F	20	*FANCD2*	c.1777C>T	p.Pro593Ser	33	UBC	No family history reported	5.80%
VUS-16 P-55	F	36	*FANCI*	c.3812C>T	p.Ser1271Phe	34	UBC	Fath ProstC	24.00%
VUS-17 P-56	F	50	*ATM*	c.7503T>A	p.Asn2501Lys	34	UBC	2 Pat Aunts BRC	35.20%
VUS-18 P-57	F	12	*SLX4*	c.4423A>G	p.Thr1475Ala	35	UBC	Pat Uncle LUC (62)	5.20%
VUS-19 P-58	F	7	*FANCB*	c.1480A>G	p.Thr494Ala	36	UBC	No family history reported	5.00%
VUS-20 P-59	F	4	*CHEK2*	c.480A>G	p.Ile160Met	36	UBC	Moth BRC (57)	12.10%
VUS-21 P-60	F	7	*ATR*	c.1602G>C	p.Trp534Cys	39	UBC	Moth BRC (48)	17.70%
VUS-22 P-61	F	4	*SMARCA4*	c.403C>G	p.Pro135Ala	40	BBC	Pat Aunt ThroatC (63); Pat uncle LiverC (63)	5.00%
VUS-23^c^ P-13	F	13	*MUTYH*	c.1258C>A	p.Leu420Met	40	OC&BC	Moth OVC (50); Pat G.Moth LUC; Sis OVC	99.70%
VUS-24 P-62	F	4	*PALB2*	c.833_834delTAinsAT	p.Leu278His	41	UBC	Sis BRC (57); Mat Aunt BRC (60)	13.40%
VUS-25 P-63	F	1	*MC1R*	c.104G>A	p.Cys35Tyr	43	BBC	No family history reported	4.70%
VUS-26^a,c^ P-22	F	12	*CHEK2*	c.1312G>T	p.Asp438Tyr	44	UBC	Sis BRC (51); Mat G.Moth BRC (87)	9.70%
VUS-27^a,c^ P-23	F	12	*CHEK2*	c.1312G>T	p.Asp438Tyr	51	UBC	Sis BRC (44); Mat G.Moth BRC (87)	9.20%
VUS-28^c^ P-39	F	15	*SLX4*	c.5413T>C	p.Cys1805Arg	45	UBC	Moth BRC (57); Sis BRC (34); Mat Aunt UterC (52)	42.00%
VUS-29^b^ P-64	F	3	*SDHB*	c.269G>A	p.Arg90Gln	46	BBC	No family history reported	2.50%
VUS-30^b^ P-64	F	13	*PALB2*	c.3428T>A	p.Leu1143His	46	BBC	No family history reported	2.50%
VUS-31^c^ P-34	F	11	*MLH1*	c.954C>A	p.His318Gln	49	UBC	Sis BRC (38); Pat Aunt BRC (70); Mat G. Moth BRC	34.20%
VUS-32 P-65	F	8	*MSH6*	c.3727A>T	p.Thr1243Ser	51	BBC	No family history reported	1.70%
VUS-33 P-66	F	4	*ATR*	c.992A>G	p.Asp331Gly	53	UBC	Sis BRC (45 & 55)	20.80%
VUS-34 P-67	F	7	*MUTYH*	c.553C>T	p.Arg185Trp	54	UBC	BRC in 1st degree relative	10.30%
VUS-35 P-68	F	8	*PALB2*	c.2821A>G	p.Ile941Val	55	BBC	Mat Cous BRC; Pat Cous BRC	1.90%
VUS-36 P-69	F	19	*BRCA1*	c.5191G>A	p.Glu1731Lys	55	UBC	BRC in 1st degree relative	8.40%
VUS-37 P-70	F	13	*MUTYH*	c.1258C>A	p.Leu420Met	55	BBC	Moth BRC (55)	5.50%

Complete patient and variant information and family history for identified variants of unknown significance (VUS).

*AOD* age of diagnosis, *BRC* breast cancer, *OVC* ovarian cancer, *UterC* uterine cancer, *StomC* stomach cancer, *ProsC* prostate cancer, *ThroatC* throat cancer, *LUC* lung cancer, *LiverC* liver cancer, *EosaphC* esophagus cancer, *GastC* gastric cancer, *ColorecC* colorectal cancer *Moth* mother, *Fath* father, *Sis* sister, *Bro* brother, *Mat* maternal, *Pat* paternal, *G* grand, *P* patient.

^a^Sibling pairs.

^b^Two VUS were identified in the same patient.

^c^Patient also had a PATH/LPATH variant.

### Patients with two variants

We also found ten patients with two variants; five patients had one likely pathogenic variant and one VUS, and another five had two VUSs. Four of the patients reported cancer in both maternal and paternal relatives, and two did not report a family history. The other four patients reported cancer only in their maternal relatives, but two of these patients were sisters (Supplemental Table 1).

### Patients and genes with no variant and healthy controls

We did not find any VUSs, pathogenic variants, or likely pathogenic variants in six breast cancer patients, and they only had benign/likely benign variants (Supplemental Table 1). Breast cancer in these patients could have been due to environmental factors or possibly genomic structural changes such as large deletions, duplications, and inversions, which were not investigated in this study. Additionally, none of the 76 patients had a VUS, pathogenic, or likely pathogenic variant in any of the following 37 genes: *AIP, BAP1, CDC73, CDK4, CDKN1B, CDKN2A, CTC1, CYLD, EPCAM, ERCC1, ERCC3, FAN1, FANCG, FLCN, GALNT12, GATA2, HOXB13, HRAS, MAX, NHP2, PDGFRA, POT1, PRKAR1A, RAD51C, RAD51D, RUNX1, SDHA, SDHAF2, SMAD4, SMARCB1, SMARCE1, SUFU, TERC, TMEM127, TP53, VHL*, and *XPA*.

Three of the four healthy controls were a 92-year-old grandmother and her 72-year-old daughter with no family history of cancer and her 50-year-old grandson with two paternal aunts with breast cancer at ages 75 and 76. The other healthy control was an unrelated 50-year-old female had no family history of cancer. The testing of the 92-year-old grandmother and her 72-year-old daughter showed no pathogenic variants or VUSs, yet the tested 50-year-old grandson had *CHEK2* (p.Arg145Trp) likely pathogenic variant, evidently from his paternal side as two of his paternal aunts had breast cancer. The other healthy female control, who was unrelated to the rest of the healthy controls, had no pathogenic variants or VUSs.

### Age distribution of variants

**Fig. 1:**
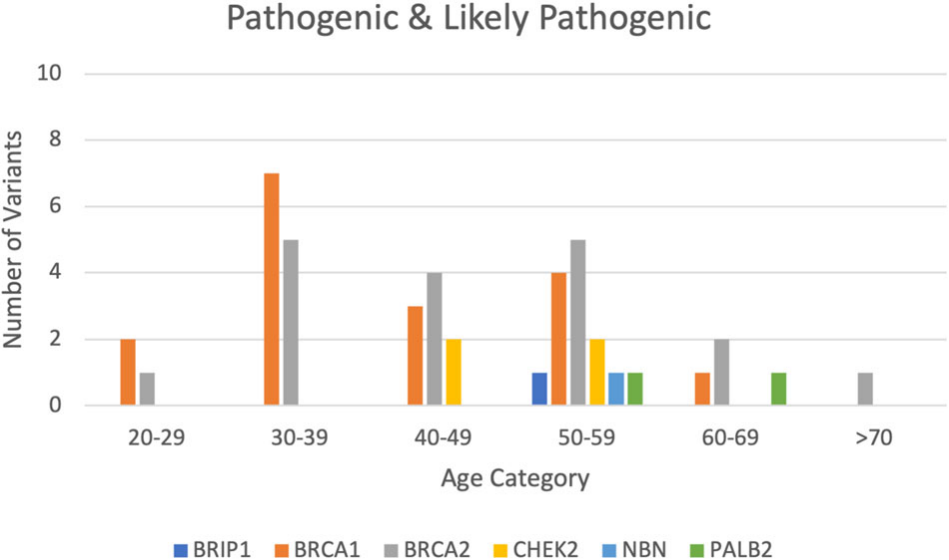
Age distribution of pathogenic and likely pathogenic variants identified in BRCA1, BRCA2, and the other genes.

We analyzed the age distribution of patients with path/LPath variants. The average age for the Path/LPath group was almost 46 years old. This group had more *BRCA1* variants in <40-year-old patients than *BRCA2* variants, and these variants were present more often in older populations (up to 70 years old). Interestingly, the non-*BRCA* variants present in this group were found mostly in the 40- and 50-year-old patients. The average age for *BRCA1* variants was 41 years (range: 26–65 years), and the average age for *BRCA2* variants was 48 years (range: 29–81 years). The average age for other variants was 50 years (range: 34–68 years) (Fig. 1).

### FATHMM, SIFT, PolyPhen2, and CADD

We used FATHMM, SIFT, PolyPhen2, and CADD prediction programs to assess the functional effects of Path/LPath classifications (Supplementary Table 2) and the VUS classifications (Table 3). One variant (*FANCD2* p.Pro593Ser) had a tolerated classification from all four prediction programs, and two other variants (*ATR* p.Asp331Gly and *AXIN2* p.Ala695Ser) were classified as tolerated by the 3 out of 4 programs. Nine of the VUSs had a FATHMM prediction as tolerant, while they had damaging predictions from the other three programs, with the exception of three, had another tolerant prediction (Table 3). These programs use different algorithms; thus, we consider all of the designations significant. All predictions for the Path/LPath variants, except variant p.Val875Leu in *BRCA2*, were damaging (D) or probably damaging (PD) with significant scores (Supplementary Table 2). Notably, one of the VUS mutations was *SLX4* c.5413T>C (p.Cys1805Arg) variant, which has been shown to disrupt *SLX4*-*SLX1* complex formation in functional studies^10^. The FATHMM classification of this variant was tolerant, yet it had a significant score from the other three programs and was consequently predicted to be damaging. We observed the same pattern in three variants classified as a VUS (*ATR* c.1602G>C, p.Trp534Cys; *PALB2*, c.2821A>G, p.Ile941Val; and *PALB2* c.3428T>A, p.Leu1143His) (Table 3). Such a comparison could be used for further assessments of variants classified as a VUS. All the variants with a FATHMM classification of a negative score had a risk estimate of being damaging, which indicates that these substitutions are likely to interfere with the function of the protein. Almost all the variants that we classified as a VUS had scores as low as the known pathogenic variants for the SIFT, PolyPhen2, and CADD programs. The variant c.838G>A (p.Ala280Thr) in the *WRAP53* gene, which we classified as a VUS, had a damaging or probably damaging score from all the programs. This variant has not been reported in breast cancer, but according to this analysis, it could interfere with the function of the protein.

**Table 3:** Comparison of FATHMM, SIFT, PolyPhen2, and CADD predications and scores for VUS.

Genes	DNA change	Protein change	FATHMM	FATHMM score	SIFT	SIFT score	PolyPhen	PolyPhen score	CADD	CADD score
*ATM*	c.3371A>T	p.Tyr1124Phe	PD	−0.58	D	0.03	D	0.912	D	22
*ATM*	c.7503T>A	p.Asn2501Lys	D	−1.98	D	0.01	D	0.974	D	25.2
*ATR*	c.992A>G	p.Asp331Gly	T	−0.87	T	0.43	T	0	PD	16.87
*ATR*	c.1602G>C	p.Trp534Cys	T	2.82	T	0.16	D	0.986	D	30
*AXIN2*	c.2083G>T	p.Ala695Ser	T	−0.1	T	0.2	T	0.2	D	22.3
*BRCA1*	c.5191G>A	p.Glu1731Lys	D	0.62	D	0.01	PD	0.767	D	32
*BRCA1*	c.5360G>A	p.Cys1787Tyr	D	−1.25	D	0	PD	0.995	D	25.8
*BRCA2*	c.8699A>T	p.Asp2900Val	PD	−0.57	D	0	D	0.999	D	27.9
*CHEK2*	c.1312G>T	p.Asp438Tyr	PD	−0.17	D	0	D	0.965	D	27
*CHEK2*	c.480A>G	p.Ile160Met	D	−3.02	D	0.01	D	0.979	D	22.6
*FANCB*	c.1480A>G	p.Thr494Ala	T	0.9078	D	0.04	PD	0.894	D	22.6
*FANCD2*	c.1777C>T	p.Pro593Ser	T	0.97	T	1	T	0	T	0.036
*FANCI*	c.3812C>T	p.Ser1271Phe	D	−1.77	D	0	PD	0.843	D	29.5
*MC1R*	c.104G>A	p.Cys35Tyr	T	0.04	PD	0.07	D	0.998	D	23.8
*MLH1*	c.954C>A	p.His318Gln	PD	−1.37	D	0	D	0.985	PD	14.82
*MSH6*	c.3727A>T	p.Thr1243Ser	D	−2.04	PD	0.06	D	0.946	D	21.3
*MUTYH*	c.553C>T	p.Arg185Trp	T	2.56	D	0.03	T	0.321	D	21.9
*MUTYH*	c.1258C>A	p.Leu420Met	D	−2.8	D	0.02	T	0.261	D	22.6
*PALB2*	c.2821A>G	p.Ile941Val	T	1.53	D	0.01	D	0.999	D	25.6
*PALB2*	c.3428T>A	p.Leu1143His	T	1.59	D	0	D	0.995	D	26.7
*POLE*	c.5653G>A	p.Ala1885Thr	D	0.9671	T	0.26	B	0.058	D	21.9
*RECQL4*	c.2704C>T	p.Arg902Trp	D	0.7973	D	0	D	0.995	D	24.8
*SDHB*	c.269G>A	p.Arg90Gln	D	−5.35	D	0	D	1.0	D	32
*SLX4*	c.421G>T	p.Gly141Trp	T	5.2	D	0	PD	0.76	D	20.3
*SLX4*	c.2320G>T	p.Ala774Ser	T	0.42	D	0	D	0.999	D	27.7
*SLX4*	c.4423A>G	p.Thr1475Ala	T	5.22	PD	0.06	T	0.024	PD	11.85
*SLX4*	c.5413T>C	p.Cys1805Arg	T	3.62	D	0	D	0.999	D	23.4
*SMARCA4*	c.403C>G	p.Pro135Ala	D	−2.16	PD	0.07	T	0.001	PD	12.31
*TSHR*	c.202C>T	p.Pro68Ser	D	−3.28	T	0.1	D	0.973	D	23.1
*WRAP53*	c.838G>A	p.Ala280Thr	PD	−0.21	D	0	D	0.988	D	28
*XRCC2*	c.268C>T	p.Leu90Phe	T	1.07	D	0.05	T	0.224	D	22.5

Scores from FATHMM, SIFT, PolyPhen, and CADD predications are compared for variants of unknown significance (VUS).

*D* damaging, *T* tolerated, *PD* probably damaging.

## Discussion

In this study, we reported 44 variants that are responsible for hereditary breast cancer and 37 VUSs that may be involved in hereditary and/or early onset of breast cancer in the Armenian population. The pathogenic/likely pathogenic group had 45 variants, 33 of which were unique, indicating that focusing on so-called founder mutations will miss the majority of the pathogenic/likely pathogenic variants causing breast cancer. We also reported 36 VUSs (31 unique), which were found in functional domains and mostly in mutational hotspots with the potential to interfere with protein function. These variants were found in known cancer-causing genes, such as *ATR*, *BRCA1*, *CHEK2*, *MLH1, and MUTYH*, and in two less prominent genes, *SLX4* and *WRAP53*. The *SLX4* complex is required for the efficient repair of DNA interstrand crosslinks (ICLs)^14^. The importance of *SLX4* for ICL repair was underscored by the findings that biallelic mutations in *SLX4* in humans cause Fanconi anemia (FA)^15^. The *SLX1*-*SLX4* complex has a preference for 5’-flap structures and promotes symmetrical cleavage of static and migrating Holliday junctions (HJs). Finally, Wilson et al. reported that *SLX1* foci could not be detected when they overexpressed a mutant form of *SLX4* (p.Cys1805Arg) that is incapable of interacting with *SLX1*^16^. The Variant *SLX4* p.Cys1805Arg allele frequency in GnomAD exomes is 0.000008, which does not exceed the estimated maximal expected allele frequency for a pathogenic SLX4 variant of 0.0001, and the variant was not found in GnomAD genomes (PM2 Pathogenic Moderate). Pathogenic predictions from several programs support its deleterious effect (PP3 Pathogenic Supporting). Variant *CHEK2* p.Asp438Tyr is in a hotspot region of 7 pathogenic nonsense and frameshift variants (PM1 Pathogenic Moderate). It has been shown to exhibit a 70% reduction in kinase activity in a cell-based assay when compared to that of wild-type *CHEK2*^17^, suggesting that the variant may have a functional impact. However, it is unclear whether such a reduction in kinase activity would be sufficient to contribute to cancer risk. The allele frequencies of this variant in European and Finnish subpopulations are slightly higher than the estimated maximal expected allele frequency of a pathogenic *CHEK2* variant (0.0000284), suggesting that this variant is likely a benign polymorphism. However, population data in this region of *CHEK2* are not considered reliable due to high pseudogene homology^18^. The variant has been reported in breast, ovarian, and prostate cancer patients in the literature, without strong evidence for causality. However, one case–control study reported a significant association with prostate cancer^19^. It was reported as a VUS in male breast cancer^20^. In our study, this variant was found in two sisters with breast cancer at ages 44 and 51, and both carry the *BRCA2* p.Val875Leu likely pathogenic variant. Variant *MLH1* p.His318Gln is in a mutation hotspot of 22 pathogenic nonsense and frameshift variants (PM1 Pathogenic Moderate). This variant is not reported in either GnomAD exomes or in GnomAD genomes (PM2 Pathogenic Moderate). A total of 207 out of 273 non-VUS missense mutations in the *MLH1* gene are pathogenic (PP2 Pathogenic Supporting). Pathogenicity prediction software does not agree on the potential impact of this missense change. The variant is reported in ClinVar as a VUS (Variation ID: 187486). This variant has not been reported in the literature in individuals with *MLH1*-related disease. The *MLH1* p.His318Gln variant was identified in a patient (i.e., P-34) who also had the LPath variant p.Ala2951Ser in the *BRCA2* gene. Since the evidence on the contribution of pathogenic variants in mismatch repair genes for breast cancer is not very strong, it is more likely that the LPath variant in *BRCA2* plays a crucial role in the development of familial breast cancer, and the involvement of the variant p.His318Gln in the *MLH1* gene in the patient’s breast cancer may be marginal or limited.

Variant *MUTYH* p.Leu420Met is in a mutation hotspot along with 12 pathogenic variants (PM1 Pathogenic Moderate). Additionally, 61 of 104 non-VUS missense mutations in the *MUTYH* gene were pathogenic (PP2 Pathogenic Supporting). The variant was observed in ExAC with an allele frequency of 71/120676 (1/1699), which does not exceed the estimated maximal expected allele frequency for a pathogenic *MUTYH* variant of 1/219. Pathogenic predictions from multiple programs support its deleterious effect (PP3 Pathogenic Supporting). This variant has been reported in a family with HNPCC and breast, pancreatic, and colorectal cancers. A functional study indicated that the variant acted comparable to wild-type^21^. Meanwhile, *MUTYH* heterozygous or homozygous mutations among breast cancer patients with or without a history of the disease evidenced an association of *MUTYH* with an increased risk of BC^22^. The *MUTYH* p.Leu420Met variant was identified in two patients (i.e., P-13 and P-70). Patient 13 also carried the LPath variant c.4358-2A>G in the *BRCA1* gene, which could play a more crucial role in the development of this patient’s breast cancer. Therefore, the role of the *MUTYH* p.Leu420Met variant may also be marginal.

Variant p.Trp1815Ter in the *BRCA1* gene was found in three unrelated female patients who were 26, 29, and 38 years old. In contrast, the male patient who also had this variant was 65 years old, a late onset for this variant in male patients. Variant p.Ile1159Metfs was found in two sets of siblings, two sisters at ages 31 and 32, and a brother/sister pair at ages 54 and 57. This difference in the age of onset for these two sibling pairs suggests a variable penetrance for this mutation. Perhaps the twin sisters had another genetic or environmental contributing factor for their early onset of breast cancer. Variant p.Leu1669Ter in *BRCA2* was also seen in two sisters with breast cancer at ages 26 and 56; it was also seen in an 81-year-old female. This large age gap between these three patients is another example of deleterious mutations in a *BRCA* gene that manifest variable penetrance.

During analysis of the age distribution for variants, we realized that more *BRCA1* and *BRCA2* variants were classified in the pathogenic group. This was expected since these two genes are studied incomparably more than others and were the first genes to be tested for HBOC. This study and similar studies emphasize the significance of comprehensive gene panels to identify non-*BRCA* variants that could be involved in hereditary breast cancer. The percentage of found VUSs using a comprehensive cancer panel with 127 genes did increase the number of VUSs, and it allowed us to find several variants implicated in hereditary cancers, 8 of which have not been reported in suspected HBOC patients. VUS rates have been recently reported as 34.8% in a combined study conducted in Greece, Turkey, and Romania using a 36-gene panel^23^. Testing of 127 genes resulted in the identification of a 45% VUS rate, which is higher than the reported rate in the recent study where a 36-gene panel was used. However, this rate is quite reasonable, considering a threefold increase in the number of genes tested compared to the currently used panels.

In conclusion, our study identified variants involved in breast cancer in the Armenian population. We also reported nine novel variants (Tables 1 and 2) that, to the best of our knowledge, had not been reported previously in patients with breast cancer. We realized that variants with a higher frequency or possible founder mutations represented only 10% of the variants, thereby missing the rest. Thus, we concluded that testing with comprehensive cancer panels increases the chances of finding cancer-causing variants in genes that are not routinely tested for in breast cancer patients. These patients and perhaps their family members would need genetic counseling before and after testing to help them understand their treatment and prevention measures, such as surgical intervention, targeted therapy, and surveillance strategies.

## Supplementary information

Supplementary Table 2

Supplementary Table for all variants

Supplementary Materials for Variant Classificaiton
