# Supplementary material for: Germline mutational spectrum in Armenian breast cancer patients suspected of hereditary breast and ovarian cancer
Source: Hum Genome Var. 2021 Feb 9;8:9. doi: 10.1038/s41439-021-00140-2 (PMC7870655; doi:10.1038/s41439-021-00140-2)
Supplement: Supplementary file 1 — Supplementary Table 2 [file 41439_2021_140_MOESM1_ESM.docx]

Supplementary Table 2- Comparison of FATHMM, SIFT, PolyPhen2, and CADD predications and scores for variants with Path/LPath classification

| Gene | DNA change | Protein change | Variant Classification | FATHMM | FATHMM score | SIFT | SIFT score | PolyPhen | PolyPhen Score | CADD | CADD Score |
| --- | --- | --- | --- | --- | --- | --- | --- | --- | --- | --- | --- |
| BRCA1 | c.211A>G | p.Arg71Gly | PATH | D | -3.11 | D | 0 | D | 0.988 | D | 33 |
| BRCA2 | c.2623G>C | p.Val875Leu | LPATH | T | 0.73 | T | 0.12 | T | 0.025 | T | 6.78 |
| BRCA2 | c.8851G>T | p.Ala2951Ser | LPATH | PD | -0.34 | D | 0 | D | 1.0 | D | 25.5 |
| CHEK2 | c.422A>C | p.Lys141Thr | LPATH | T | 4.78 | D | 0.04 | PD | 0.835 | D | 26.2 |
| CHEK2 | c.470T>C | p.Ile157Thr | LPATH | D | -2.05 | D | 0.02 | PD | 0.514 | D | 23.8 |
| CHEK2 | c.499G>A | p.Gly167Arg | LPATH | D | -5.31 | D | 0 | D | 1.0 | D | 28.6 |
| SDHB | c.269G>A | p.Arg90Gln | LPATH | D | -5.35 | D | 0 | D | 1.0 | D | 32 |

Scores from FATHMM, SIFT, PolyPhen, and CADD predications are compared for pathogenic/likely pathogenic and variants of unknown significance (VUS). PATH= pathogenic; LPATH= likely pathogenic; D= damaging; T= tolerated; PD= probably damaging.
