# Supplementary material for: Germline mutational spectrum in Armenian breast cancer patients suspected of hereditary breast and ovarian cancer
Source: Hum Genome Var. 2021 Feb 9;8:9. doi: 10.1038/s41439-021-00140-2 (PMC7870655; doi:10.1038/s41439-021-00140-2)
Supplement: Supplementary file 3 — Supplementary Materials for Variant Classificaiton [file 41439_2021_140_MOESM3_ESM.pdf]

Supplementary Materials for Germline Mutational Spectrum in the Armenian Hereditary Breast and Ovarian Cancer Patients

Detailed description of all variant annotations.

The BRCA1 and BRCA2 Pathogenic or Likely Pathogenic Variants

BRCA1 (NM_007299.4: c.211A>G; p.Arg71Gly)– The *BRCA1* variant p.Arg71Gly is a known pathogenic missense mutation in exon 5 that is found in the Zinc finger domain, RING/FYVE/PHD-type (E10-85E aa). It is now recognized to bind DNA, RNA, protein and/or lipid substrates (Gamsjaeger et al., 2007). It is found in a mutational hotspot including 28 pathogenic frameshift and nonsense variants (PM1 Pathogenic Moderate). It was conformed via RT-PCR that this variant results in the loss of 22 nucleotides in exon 5 and a subsequent premature stop codon due to a cryptic splice donor site (Vega et al., 2001; Santos et al., 2009) (PS3 Pathogenic Strong). The allele frequency in GnomAD exomes is 0.000004 which does not exceed the estimated maximal expected allele frequency for a pathogenic BRCA1 variant of 0.0001, and the variant is not found in GnomAD genomes (PM2 Pathogenic Moderate). Three different pathogenic missense changes at the same amino acid residue (chr17:41258473C>A (Arg71Ile); chr17:41258473C>G (Arg71Thr); chr17:41258473C>T (Arg71Lys)) have been reported in ClinVar (PM5 Pathogenic Moderate). 10 pathogenic predictions from DANN, EIGEN, FATHMM-MKL, M-CAP, MVP, MutationAssessor, MutationTaster, PrimateAI, REVEL and SIFT versus 1 benign prediction from DEOGEN2 support its deleterious effect (PP3 Pathogenic Supporting). The variant has been classified as pathogenic by the ClinGen-approved ENIGMA expert panel (ClinVar SCV001161539.1) (PP5 Pathogenic Supporting). In our study this variant was found in a 36-year-old female with unilateral breast cancer and a family history of breast cancer.

BRCA1 (NM_007294.3: c.302-1G>A)– The intronic/splice site variant c.302-1G>A is just before exon 7 sequence coding for Y101-147L aa. The closest functional domain is *BRCA1*, serine-rich domain (A344-507R). This splice site variant could result in a truncated or altered protein, potentially interfering with its function in DNA repair as an established disease mechanism in hereditary breast and ovarian cancer (PVS1 Pathogenic Very Strong). The variant is not found in GnomAD exomes neither in GnomAD genomes (PM2 Pathogenic Moderate). Several variants have been reported in this splice site either at position c.302-1 or c.302-2 (source, ClinVar). 4 pathogenic predictions from DANN, EIGEN, FATHMM-MKL and MutationTaster versus no benign predictions support its deleterious effect (PP3 Pathogenic Supporting). This variant was previously reported in ClinVar in patients diagnosed with Hereditary breast and ovarian cancer syndrome. In our study this variant was found in a 32-year-old female with unilateral breast cancer and no reported family history. Based on the available data, we classified this variant as a Pathogenic.

BRCA1 (NM_007294.3: c.798_799del; p.Ser267Lysfs)– The BRCA1 p.Ser267Lysfs is a known pathogenic frameshift variant in exon 11 in a non-functional domain just before the serine-rich domain (A344-507R aa) and many other downstream domains. This null variant (frame-shift) is predicted to encode a truncated non-functional protein, and heterozygous loss of function of the BRCA1 gene is an established disease mechanism in hereditary breast and ovarian cancer (PVS1 Pathogenic Very Strong). It is found in a mutational hotspot of 29 pathogenic variants (PM1 Pathogenic Moderate). The variant is not found in GnomAD exomes neither in GnomAD genomes (PM2 Pathogenic Moderate). The variant has been classified as pathogenic by the ClinGen-approved ENIGMA expert panel (ClinVar SCV00028235.01) (PP5 Pathogenic Supporting). 1 pathogenic prediction from GERP versus no benign prediction supports its deleterious effect (PP3 Pathogenic Supporting). In this study this variant was found in a 56-year- old female with unilateral breast cancer and a family history of cancer.

BRCA1 (NM_007294.3: c.1504_1508del; p.Leu502Alafs)– The *BRCA1* p.Leu502Alafs is a known pathogenic frameshift variant in exon 11 and in the serine-rich domain (A344-507R aa) with limited information, however there's information about the SQ (serine-glutamine rich) cluster, whose phosphorylation is critical for allowing adequate time for completing normal Homologous Recombination Repair (HRR) prior to mitosis and preventing cells from entering G1 prematurely resulting in gross chromosomal aberrations (Beckta et al., 2015). The deletion causes a frameshift, which changes a Leucine to an Alanine at codon 502, and creates a premature stop codon at position 2 of the new reading frame, and this null variant is predicted to cause loss of normal protein function through either protein truncation or nonsense-mediated mRNA decay which is an established disease mechanism in hereditary breast and ovarian cancer (PVS1 Pathogenic Very Strong). This variant is also in a mutational hotspot of 32 pathogenic frameshift and nonsense variants (PM1 Pathogenic Moderate). The allele frequency in GnomAD exomes is 0.0000119 which does not exceed the estimated maximal expected allele frequency for a pathogenic BRCA1 variant of 0.0001, and this variant is not found in GnomAD genomes (PM2 Pathogenic Moderate). The variant has been classified as pathogenic by the ClinGen-approved ENIGMA expert panel (ClinVar SCV000282260.1) (PP5 Pathogenic Supporting). 1 pathogenic prediction from GERP versus no benign prediction supports its deleterious effect (PP3 Pathogenic Supporting). In this study this variant was seen in a 40 years old female with bilateral breast cancer and no reported family history of cancer.

BRCA1 (NM_007294.3: c.2649_2650insGGCA; p.Thr884Glyfs)– The BRCA1 p.Thr884Glyfs is a known pathogenic variant in exon 11 in a non-functional domain just before the BRSTCANCERI domain (S1180-1200Q aa) (Xu et al., 1999) and in a mutational hotspot with 37 pathogenic variants (PM1 Pathogenic Moderate). This frameshift variant truncates the protein and potentially hinder HRR as an established disease mechanism in hereditary breast and ovarian cancer (PVS1 Pathogenic Very Strong). The variant is not found in GnomAD exomes neither in GnomAD genomes (PM2 Pathogenic Moderate). The variant has been classified as pathogenic by the ClinGen-approved ENIGMA expert panel (ClinVar SCV000299802.2) (PP5 Pathogenic Supporting). In this study this variant was found in a 36-year-old female with unilateral breast cancer and a family history of cancer.

BRCA1 (NM_007294.3: c.3436_3439del; p.Cys1146Leufs)– This *BRCA1* is a known pathogenic variant in exon 11 in a non-functional domain just before the BRSTCANCERI domain (S1180-1200Q aa) (Xu et al., 1999) and in a mutational hotspot with 27 pathogenic variants (PM1 Pathogenic Moderate). The deletion causes a frameshift, which changes a Cysteine to a Leucine at codon 1146, and introduces a premature stop codon at position 8 of the new reading frame. This variant is predicted to cause loss of normal protein function either through protein truncation or nonsense-mediated mRNA decay which is an established disease mechanism in hereditary breast and ovarian cancer (PVS1 Pathogenic Very Strong). The variant is not found in GnomAD exomes neither in GnomAD genomes (PM2 Pathogenic Moderate). The variant has been classified as pathogenic by the ClinGen-approved ENIGMA expert panel (ClinVar SCV000299942.2) (PP5 Pathogenic Supporting). 1 pathogenic prediction from GERP versus no benign prediction supports its deleterious effect (PP3 Pathogenic Supporting). In this study the variant Cys1146Leufs was found twice in a 32 and a 45- year-old female with unilateral breast cancer and family history of breast cancer, respectively.

BRCA1 (NM_007294.3: c.3477_3480del; p.Ile1159Metfs)– This *BRCA1* is a known pathogenic variant also in exon 11 in a non-functional domain just before the BRSTCANCERI domain (S1180-1200Q aa) (Xu et al., 1999) and in a mutational hotspot with 35 pathogenic variants (PM1 Pathogenic Moderate). The deletion causes a frameshift, which changes an Isoleucine to a Methionine at codon 1159, and creates a premature stop codon at position 50 of the new reading frame. This null variant is

predicted to cause loss of normal protein function through either protein truncation or nonsense-mediated mRNA decay which is an established disease mechanism in hereditary breast and ovarian cancer (PVS1 Pathogenic Very Strong). The allele frequency in GnomAD exomes is 0.00000398 which does not exceed the estimated maximal expected allele frequency for a pathogenic BRCA1 variant of 0.0001, and the variant is not found in GnomAD genomes (PM2 Pathogenic Moderate). The variant has been classified as pathogenic by the ClinGen-approved ENIGMA expert panel (ClinVar SCV000299950.2) (PP5 Pathogenic Supporting). 1 pathogenic prediction from GERP versus no benign prediction supports its deleterious effect (PP3 Pathogenic Supporting). In this study the variant p.Ile1159Metfs was found in 4 patients from two families– a sister/sister and a brother/sister pair (PP1 Pathogenic Supporting). The two sisters were 31 and 32 years old and the brother/sister pair were 57 and 54, respectively; all had unilateral breast cancer.

BRCA1 (NM_007294.3: c.3485delA; p.Asp1162Valfs)– This *BRCA1* is a known pathogenic variant also in exon 11 in a non-functional domain just before the BRSTCANCERI domain (S1180-1200Q aa) (Xu et al., 1999) and in a mutational hotspot with 34 pathogenic variants (PM1 Pathogenic Moderate). The deletion causes a frameshift, which changes an Aspartic Acid to a Valine at codon 1162, and creates a premature stop codon at position 48 of the new reading frame. This variant is predicted to cause loss of normal protein function through either protein truncation or nonsense-mediated mRNA decay which is an established disease mechanism in hereditary breast and ovarian cancer (PVS1 Pathogenic Very Strong). The allele frequency in GnomAD exomes is 0.00000398 which does not exceed the estimated maximal expected allele frequency for a pathogenic BRCA1 variant of 0.0001, and the variant is not found in GnomAD genomes (PM2 Pathogenic Moderate). The variant has been classified as pathogenic by the ClinGen-approved ENIGMA expert panel (ClinVar SCV000299952.2) (PP5 Pathogenic Supporting). 1 pathogenic prediction from GERP versus no benign prediction supports its deleterious effect (PP3 Pathogenic Supporting). In this study the variant Asp1162Valfs was found in a 51- year-old female with unilateral breast cancer and strong family history.

BRCA1 (NM_007294.3: c.4358-2A>G)– This variant was found in the splice junction of exon 13 of the *BRCA1* gene. Based on the position of this variant, a splice site receptor, it can potentially interfere with splicing which is an established disease mechanism (PVS1 Pathogenic Very Strong). The variant is not found in GnomAD exomes neither in GnomAD genomes (PM2 Pathogenic Moderate). 3 pathogenic predictions from EIGEN, FATHMM-MKL and MutationTaster versus 1 benign prediction from DANN support its deleterious effect (PP3 Pathogenic Supporting). In this study this variant was found in a female patient with unilateral breast cancer and ovarian cancer at age 40 and with a strong family history of cancer (PP4 Pathogenic Supporting). Therefore, this variant was classified as a Likely Pathogenic.

BRCA1 (NM_007294.3: c.5444G>A; p.Trp1815Ter)– The *BRCA1* variant p.Trp1815Ter is a known pathogenic nonsense variant in exon 23 and in the functional domain of BRCT2 (aa 1756-1855). *BRCA1* contains at its C terminus two copies of a conserved domain that was named BRCT for *BRCA1* C terminus. This domain of about 95 amino acids is found in a large variety of proteins involved in DNA repair, recombination and cell cycle control (Bork et al., 1997). This null (nonsense) variant is predicted to encode a truncated non-functional protein which is an established disease mechanism in hereditary breast and ovarian cancer (PVS1 Pathogenic Very Strong). This variant was observed in a mutation hotspot region of 21 pathogenic variants (source, ClinVar) (PM1 Pathogenic Moderate). The variant is not found in GnomAD exomes neither in GnomAD genomes (PM2 Pathogenic Moderate). The variant has been classified as pathogenic by the ClinGen-approved ENIGMA expert panel (ClinVar SCV000300259.2) (PP5 Pathogenic Supporting). 4 pathogenic predictions from DANN, EIGEN,

FATHMM-MKL and MutationTaster versus no benign predictions support its deleterious effect (PP3 Pathogenic Supporting). In this study this variant was seen in 4 unrelated patients with a unilateral breast cancer and a strong family history of breast cancer.

BRCA2 (NM_000059.3: c.574dupA; p.Met192Asnfs)– The *BRCA2* variant p.Met192Asnfs is a known pathogenic variant in exon 7 in a non-functional domain. The location of this frameshift variant is significant since it could potentially interfere with the function of almost the entire protein which is an established disease mechanism in hereditary breast and ovarian cancer (PVS1 Pathogenic Very Strong). This variant was observed in a mutation hotspot region including 18 pathogenic variants (source, ClinVar) (PM1 Pathogenic Moderate). The variant is not found in GnomAD exomes neither in GnomAD genomes (PM2 Pathogenic Moderate). 1 pathogenic prediction from GERP versus no benign prediction supports its deleterious effect (PP3 Pathogenic Supporting). The variant has been classified as pathogenic by the ClinGen-approved ENIGMA expert panel (ClinVar SCV000324374.1) (PP5 Pathogenic Supporting). In this study this deleterious variant was found in a 36-year-old female with bilateral breast cancer and no reported family history.

BRCA2 (NM_000059.3: c.1414C>T; p.Gln472Ter)– The *BRCA2* variant p.Gln472Ter is a known pathogenic variant in exon 10 and in a non- functional domain. This nonsense variant truncates the protein and thus makes it non-functional which is an established disease mechanism in hereditary breast and ovarian cancer (PVS1 Pathogenic Very Strong). This variant was observed in a mutation hotspot region of 20 pathogenic variants (source, ClinVar) (PM1 Pathogenic Moderate). The variant is not found in GnomAD exomes neither in GnomAD genomes (PM2 Pathogenic Moderate). The variant has been classified as pathogenic by the ClinGen-approved ENIGMA expert panel (ClinVar SCV000300429.2) (PP5 Pathogenic Supporting). In this study this variant was found in a 53-year-old female with unilateral breast cancer and a strong family history.

BRCA2 (NM_000059.3: c.1528G>T; p.Glu510Ter)– The *BRCA2* variant p.Gln472Ter is a known pathogenic variant in exon 10 and in a non-functional domain. The substitution creates a nonsense variant, which changes a Glutamic Acid to a premature stop codon (GAA>TAA), and is predicted to cause loss of normal protein function through either protein truncation or nonsense-mediated mRNA decay which is an established disease mechanism in hereditary breast and ovarian cancer (PVS1 Pathogenic Very Strong). This variant was observed in a mutation hotspot region of 20 pathogenic variants (source, ClinVar) (PM1 Pathogenic Moderate). This variant is not found in GnomAD exomes neither in GnomAD genomes (PM2 Pathogenic Moderate). 3 pathogenic predictions from DANN, EIGEN and MutationTaster versus 1 benign prediction from FATHMM-MKL support its deleterious effect (PP3 Pathogenic Supporting). The variant has been classified as pathogenic by the ClinGen-approved ENIGMA expert panel (ClinVar SCV000300437.2) (PP5 Pathogenic Supporting). In this study this variant was found in a 55-year-old female with unilateral breast cancer and a family history of cancer.

BRCA2 (NM_000059.3: c.2095C>T; p.Gln699Ter)– The *BRCA2* variant p.Gln699Ter is a known pathogenic variant in exon 11 in a non-functional domain. This nonsense variant truncates the protein and thus makes in non-functional which is an established disease mechanism in hereditary breast and ovarian cancer (PVS1 Pathogenic Very Strong). This variant was observed in a mutation hotspot region of 14 pathogenic variants (source, ClinVar) (PM1 Pathogenic Moderate). This variant is not found in GnomAD exomes neither in GnomAD genomes (PM2 Pathogenic Moderate). The variant has been classified as pathogenic by the ClinGen-approved ENIGMA expert panel (ClinVar SCV000324047.1) (PP5 Pathogenic Supporting). In this study this deleterious variant was found in a 30-year-old female with unilateral breast cancer and a family history of cancer.

BRCA2 (NM_000059.3: c.2623G>C; p.Val875Leu)– This variant is in exon 11 of the BRCA 2 gene, in the non-functional domain region (Q175-1823K aa). This variant is in a mutation hotspot region of 26 pathogenic variants (source, ClinVar) (PM1 Pathogenic Moderate). One study classified the p.Val875His variant as pathogenic, concluding that the Homology Directed Repair function of *BRCA2* could be compromised (Guidugli et al., 2018). This variant is not found in GnomAD exomes neither in GnomAD genomes (PM2 Pathogenic Moderate). 8 benign predictions from DANN, EIGEN, FATHMM-MKL, MVP, MutationTaster, PrimateAI, REVEL and SIFT versus 1 pathogenic prediction from M-CAP support its benign effect (BP4 Benign Supporting). In our study this variant was found in two sisters ages 44 and 51 with unilateral breast cancer (PP1 Pathogenic Supporting). Therefore, the evidence above suggests that this variant could be classified as a Likely Pathogenic.

BRCA2 (NM_000059.3: c.2808_2811del; p.Ala938Profs)– The *BRCA2* variant p.Ala938Profs is a known pathogenic variant in exon 11in a non-functional domain and in a mutation hotspot region of 23 pathogenic variants (PM1 Pathogenic Moderate). This frameshift variant truncates the protein domains after this residue which is an established disease mechanism in hereditary breast and ovarian cancer (PVS1 Pathogenic Very Strong). The allele frequency in GnomAD exomes is 0.00000797 which does not exceed the estimated maximal expected allele frequency for a pathogenic BRCA2 variant of 0.0001, and this variant is not found in GnomAD genomes (PM2 Pathogenic Moderate). 1 pathogenic prediction from GERP versus no benign predictions supports its deleterious effect (PP3 Pathogenic Supporting). The variant has been classified as pathogenic by the ClinGen-approved ENIGMA expert panel (ClinVar SCV000282373.1) (PP5 Pathogenic Supporting). In this study this deleterious variant was found in two patients– a 29 and a 38-year-old female with unilateral breast cancer and a family history of cancer.

BRCA2 (NM_000059.3: c.4037_4038delCT; p.Thr1346Serfs)– The *BRCA2* variant p.Thr1346Serfs is a known pathogenic variant in exon 11 in a non-functional domain just before the *BRCA2*_REPEAT (F1421-1454P aa) domain, which, with other 39 aa repeats participates in RAD51 binding (a key protein in DNA recombinational repair) and confers resistance to methyl methanesulphonate treatment. This frameshift variant disrupts the function of the downstream domains which is an established disease mechanism in hereditary breast and ovarian cancer (PVS1 Pathogenic Very Strong). This variant is in a mutation hotspot region of 21 pathogenic variants (source, ClinVar) (PM1 Pathogenic Moderate). This variant is not found in GnomAD exomes neither in GnomAD genomes (PM2 Pathogenic Moderate). The variant has been classified as pathogenic by the ClinGen-approved ENIGMA expert panel (ClinVar SCV000282386.1) (PP5 Pathogenic Supporting). In our study this variant was found in a 53-year-old female with unilateral breast cancer and a family history of cancer.

BRCA2 (NM_000059.3: c.4548_4549delCA; p.Lys1517Argfs)– The *BRCA2* variant p.Lys1517Argfs is a known pathogenic variant in exon 11 in the *BRCA2*_REPEAT domain (F1009-2083F aa) and in a mutation hotspot region of 22 pathogenic variants (source, ClinVar) (PM1 Pathogenic Moderate). This domain, with other 39 aa repeats, participates in RAD51 binding (a key protein in DNA recombinational repair) and resistance to methyl methanesulphonate treatment. This deleterious variant truncates the protein domains and distroys its function (Chen et al., 1998) (PVS1 Pathogenic Very Strong; PS3 Pathogenic Strong). This variant is not found in GnomAD exomes neither in GnomAD genomes (PM2 Pathogenic Moderate). The variant has been classified as pathogenic by the ClinGen-approved ENIGMA expert panel (ClinVar SCV000783768.1) (PP5 Pathogenic Supporting). In our study the variant p.Lys1517Argfs was found in a 29-year-old female with unilateral breast cancer and family history of cancer.

BRCA2 (NM_000059.3: c.5006T>G; p.Leu1669Ter)– The *BRCA2* variant p. Leu1669Ter is in exon 11 in the *BRCA2*_REPEAT domain (F1009-2083F aa) and in a mutation hotspot region of 21 pathogenic

variants (source, ClinVar) (PM1 Pathogenic Moderate). This domain, with other 39 aa repeats, participates in RAD51 binding (a key protein in DNA recombinational repair) and resistance to methyl methanesulphonate treatment. This deleterious variant truncates the protein domains and distroys its function (Chen et. Al, 1998) (PVS1 Pathogenic Very Strong; PS3 Pathogenic Strong). This variant is not found in GnomAD exomes neither in GnomAD genomes (PM2 Pathogenic Moderate). This variant is reported in ClinVar as a pathogenic variant. In our study the variant p.Leu1669Ter was found in 3 patients; two sisters and an unrelated female, all three with unilateral breast cancer. The sisters were 26 and 56-year-old and the third female was 81 years old, all with a family history of breast cancer. Therefore, based on abovementioned data, this variant was classified as a Pathogenic.

BRCA2 (NM_000059.3: c.6302del; p.Asn2101Metfs)– The *BRCA2* variant p.Asn2101Metfs is a known pathogenic variant in exon 11in a non-functional domain after the *BRCA2*_REPEAT (N2051-2085L aa) functional domain and in a mutation hotspot region with 31 pathogenic variants (PM1 Pathogenic Moderate). This frameshift mutation disrupts the function of the downstream BRCA-2_helical domain (D2479- 2667S aa) (Yang et al., 2002) (PVS1 Pathogenic Very Strong; PS3 Pathogenic Strong). 1 pathogenic prediction from GERP versus no benign predictions supports its deleterious effect (PP3 Pathogenic Supporting). This variant is not found in GnomAD exomes neither in GnomAD genomes (PM2 Pathogenic Moderate). The variant has been classified as pathogenic by the ClinGen-approved ENIGMA expert panel (ClinVar SCV000324438.1) (PP5 Pathogenic Supporting). In our study this pathogenic variant was found in a 31-year- old female with unilateral breast cancer and a strong family history.

BRCA2 (NM_000059.3: c.7689delC; p.His2563Glnfs)– The *BRCA2* variants p.His2563Glnfs is a known pathogenic variant in exon 16 in the BRCA-2_helical domain (D2479- 2667S aa) in a mutation hotspot of 22 pathogenic variants (PM1 Pathogenic Moderate). This domain binds the 70-amino acid DSS1 (deleted in split-hand/split-foot syndrome) protein, which was originally identified as one of three genes that map to a 1.5-Mb locus deletion in an inherited developmental malformation syndrome (Yang et al., 2002). The deletion causes a frameshift, which changes a Histidine to a Glutamine at codon 2563, and creates a premature stop codon at position 85 of the new reading frame which is an established disease mechanism in hereditary breast and ovarian cancer (PVS1 Pathogenic Very Strong). This variant is not found in GnomAD exomes neither in GnomAD genomes (PM2 Pathogenic Moderate). The variant has been classified as pathogenic by the ClinGen-approved ENIGMA expert panel (ClinVar SCV000301193.2) (PP5 Pathogenic Supporting). In this study this deleterious variant was found twice in a 68-year-old male patient and 38-year-old female patient both with unilateral breast cancer and a family history of cancer.

BRCA2 (NM_000059.3: c.8851G>T; p.Ala2951Ser)– The BRCA2 variant p.Ala2951Ser was found in two functional domains – a nucleic acid-binding OB-fold (R2669-3184L aa), which functions as ssDNA binding and nucleic acid recognition site, and the Tower domain (M2831-2967T aa) with a major role in tumor suppression and DNA binding. This variant is in a mutation hotspot with 16 pathogenic variants, some of them reported in the vicinity of residue Ala2951, and one variant (i.e., c.8850_8851dupGG (p.Ala2951Glyfs)) at the same position. This variant is not found in GnomAD exomes neither in GnomAD genomes (PM2 Pathogenic Moderate). 7 pathogenic predictions from DANN, EIGEN, FATHMM-MKL, M-CAP, MutationTaster, PrimateAI and SIFT versus 2 benign predictions from MVP and REVEL support its deleterious effect (PP3 Pathogenic Supporting). In our study this variant was found in a 49-year-old female with unilateral breast cancer and a strong family history of breast cancer. Based on the evidence above we classified this variant as a Likely Pathogenic.

BRCA2 (NM_000059.3: c.9097dupA; p.Thr3033Asnfs)– The *BRCA2* variant p.Thr3033Asnfs is a known pathogenic variant in exon 23 in the Nucleic acid-binding OB-fold (T2968-3184L aa) domain, which binds to single-stranded nucleic acids (staphylococcal nuclease and aspartyl-tRNA synthetase) or oligosaccharides (B subunits of enterotoxin and verotoxin-1), and has been termed the oligonucleotide/oligosaccharide binding motif, or OB fold (Agrawal & Kishan, 2003). This frameshift mutation disrupts the function of the domain which is an established disease mechanism in hereditary breast and ovarian cancer (PVS1 Pathogenic Very Strong). This mutation hotspot has 31 pathogenic variants (PM1 Pathogenic Moderate). This variant is not found in GnomAD exomes neither in GnomAD genomes (PM2 Pathogenic Moderate). 1 pathogenic prediction from GERP versus no benign predictions supports its deleterious effect (PP3 Pathogenic Supporting). The variant has been classified as pathogenic by the ClinGen-approved ENIGMA expert panel (ClinVar SCV000282467.1) (PP5 Pathogenic Supporting). The variant p.Thr3033Asnfs was found in a 48-year-old female with unilateral breast cancer and a strong family history.

BRCA2 (NM_000059.3: c.9253delA; p.Thr3085Glnfs)– The *BRCA2* variant p.Thr3085Glnfs is a known pathogenic variant in exon 23 in the Nucleic acid-binding OB-fold (T2968-3184L aa) domain and in a mutation hotspot of 16 pathogenic variants (PM1 Pathogenic Moderate). This frameshift variant disrupts the function of the domain (PVS1 Pathogenic Very Strong). This variant is not found in GnomAD exomes neither in GnomAD genomes (PM2 Pathogenic Moderate). 1 pathogenic prediction from GERP versus no benign predictions supports its deleterious effect (PP3 Pathogenic Supporting). The variant has been classified as pathogenic by the ClinGen-approved ENIGMA expert panel (ClinVar SCV000324753.1) (PP5 Pathogenic Supporting). In our study the variant p.Thr3085Glnfs was found in a 41-year-old female with unilateral breast cancer with a family history of cancer.

Other Pathogenic or Likely Pathogenic Variants

BRIP1 (NM_032043.2: c.917dupA; p.Asn306Lysfs)– The *BRIP1* gene pathogenic variant Asn306Lysfs is in exon 7 in the DEAD_2 domain (F248-415L aa) and in a mutation hotspot of 9 pathogenic variants (PM1 Pathogenic Moderate). This represents a conserved region within a number of RAD3-like DNA-binding helicases that are seemingly ubiquitous– members include proteins of eukaryotic, bacterial and archaeal origin. RAD3 is involved in nucleotide excision repair, and forms part of the transcription factor TFIIH in yeast. This deleterious frame shift mutation distroys the downstream *BRCA1* binding domain which is an established disease mechanism in hereditary breast and ovarian cancer (PVS1 Pathogenic Very Strong). This variant is not found in GnomAD exomes neither in GnomAD genomes (PM2 Pathogenic Moderate). 1 pathogenic prediction from GERP versus no benign predictions supports its deleterious effect (PP3 Pathogenic Supporting). This variant is reported in ClinVar as a pathogenic or a likely pathogenic variant. In our study it was found in a 59-year-old female with unilateral breast cancer and a family history of cancer. Therefore, this variant was classified as a Pathogenic.

CHEK2 (NM_007194.3: c.409C>T; p.Arg137Ter)- The CHEK2 variant p.Arg137Ter is in exon 3, in a kinase domain and in a mutation hotspot of 17 pathogenic variants (PM1 Pathogenic Moderate). This nonsense variant truncates the protein and thus makes it non-functional which is an established disease mechanism in disease (PVS1 Pathogenic Very Strong). The allele frequency in GnomAD exomes is 0.0000239 which does not exceed the estimated maximal expected allele frequency for a pathogenic CHEK2 variant of 0.0001, and this variant is not found in GnomAD genomes (PM2 Pathogenic Moderate). 4 pathogenic predictions from DANN, EIGEN, FATHMM-MKL and MutationTaster versus no benign predictions support its deleterious effect (PP3 Pathogenic Supporting). In our study this variant was found in a 54-year-old female with unilateral breast cancer and a family history. Based on

the evidence above we classified this variant as a Pathogenic.

CHEK2 (NM_007194.3: c.422A>C; Lys141Thr)– This variant is in exon 3 of the *CHEK2* gene in the forkhead-associated (FHA) domain (aa 113-175); it functions as a phosphopeptide recognition domain found in many regulatory proteins. It is in a mutation hotspot of 16 pathogenic variants (source ClinVar) (PM1 Pathogenic Moderate). This variant is not found in GnomAD exomes neither in GnomAD genomes (PM2 Pathogenic Moderate). This variant has been reported in the literature in individuals affected with breast cancer (Tung et al., 2016; Kraus et al., 2017). 12 pathogenic predictions from DANN, DEOGEN2, EIGEN, FATHMM-MKL, M-CAP, MVP, MutationAssessor, MutationTaster, REVEL, SIFT, PolyPhen-2 and Align-GVGD versus 1 benign prediction from PrimateAI support its deleterious effect (PP3 Pathogenic Supporting). In our study this variant was found in a 45-year-old female with unilateral breast cancer and a strong family history. This patient also had a VUS in the *SLX4* gene. Although this variant has been reported in ClinVar as a VUS, based on the evidence provided above, we classified this variant as a Likely Pathogenic.

CHEK2 (NM_007194.4: c.470T>C; p.Ile157Thr)– The CHEK2 variant p.Ile157Thr was observed in the kinase domain in a tight region between amino acid 407-499 and in a mutation hotspot of 13 pathogenic variants (PM1 Pathogenic Moderate). 4 functional studies (Falck et al., 2001; Kilpivaara et al., 2004; Li et al., 2002) confirmed the likely pathogenic effect of this variant (PS3 Pathogenic Strong). An alternative variant (chr22:29121087 A>C (Ile157Ser)) at the same amino acid residue is classified as likely pathogenic (PM5 Pathogenic Moderate). Meantime, majority of missense variants detected in CHEK2 are pathogenic and known cause of disease (PP2 Pathogenic Supporting). The homozygous allele count in GnomAD exomes is 11 which exceeds the estimated maximal expected count for a pathogenic CHEK2 variant of 3 (BS2 Benign Strong). In our study the variant p.Ile157Thr was found in a 46-year-old female with family history of cancer. In summary, based on the evidence and given the previous reports of the variant in association with increased risk of breast cancer, we classified this variant as a Likely Pathogenic.

CHEK2 (NM_007194.3: c.499G>A; p.Gly167Arg)– The CHEK2 variant p.Gly167Arg was observed in the kinase domain in a tight region between amino acid 407-499 and in a mutation hotspot of 13 pathogenic variants (PM1 Pathogenic Moderate). One functional study (Roeb et al., 2012) confirmed its damaging effect (PS3 Pathogenic Strong). Meantime, an equivalent variant (chr22:29121058 C>G (Gly167Arg)) at the same amino acid residue is classified as pathogenic by UniProt (PS1 Pathogenic Strong). The allele frequency in GnomAD exomes is 0.0000239 which does not exceed the estimated maximal expected allele frequency for a pathogenic CHEK2 variant of 0.0001, and the variant is not found in GnomAD genomes (PM2 Pathogenic Moderate). Meantime, majority of missense variants detected in CHEK2 are pathogenic and known cause of disease (PP2 Pathogenic Supporting). 10 pathogenic predictions from DANN, DEOGEN2, EIGEN, FATHMM-MKL, M-CAP, MVP, MutationAssessor, MutationTaster, REVEL and SIFT versus 1 benign prediction from PrimateAI support its deleterious effect (PP3 Pathogenic Supporting). In our study the variant p.Gly167Arg was found in a 54-year-old female with family history of breast cancer. In summary, based on the evidence and given the previous reports of the variant in association with increased risk of breast cancer, we classified this variant as a Likely Pathogenic.

NBN (NM_002485.4: c.1502G>A; p.Trp501Ter)– The *NBN* gene pathogenic variant p.Trp501Ter is in exon 11 and before the DNA repair Nbs1 C-terminal domain (K683-746Y aa). This C- terminal region of the DNA damage repair protein Nbs1 has been identified to be necessary for the binding of *Mre11* and *Tel1* (You et al., 2005). The substitution creates a nonsense variant, which changes a Tryptophan to a premature stop codon (TGG>TAG), and is predicted to cause loss of normal protein function through

either protein truncation which misses the *Mre11* and *ATM* binding domains or nonsense-mediated mRNA decay (PVS1 Pathogenic Very Strong). This variant is not found in GnomAD exomes neither in GnomAD genomes (PM2 Pathogenic Moderate). 3 pathogenic predictions from EIGEN, FATHMM-MKL and MutationTaster versus no benign predictions support its deleterious effect (PP3 Pathogenic Supporting). This variant is reported in ClinVar as a pathogenic or a likely pathogenic variant. In our study this variant was found in a 51-year-old female with unilateral breast cancer and a family history of cancer. Therefore, based on the evidence provided above, we classified this variant as a Pathogenic.

PALB2 (NM_024675.3: c.932_933insC; p.Lys311Asnfs)– The *PALB2* variant p.Lys311Asnfs is a frameshift variant located in exon 4 and the *BRCA1* interaction functional domain. It is expected to result in an absent or disrupted protein product and loss-of-function variants in PALB2 are known to be pathogenic (Antoniou et al., 2014; Janatova et al., 2013; Rahman et al., 2007) (PVS1 Pathogenic Very Strong). It is in a mutation hotspot in a vicinity of 8 pathogenic variants (PM1 Pathogenic Moderate). This variant is not found in GnomAD exomes neither in GnomAD genomes (PM2 Pathogenic Moderate). This variant is reported in ClinVar as a pathogenic variant. In our study this variant was found in a 68-year-old female with a family history of cancer. Particularly, her brother was diagnosed with prostate cancer at age 62. Therefore, based on the evidence, we classified this variant as a Pathogenic.

PALB2 (NM_024675.3: c.3299_3306dup; p.Val1103Leufs)– The *PALB2* variant p.Val1103Leufs is a frameshift variant in exon 12 located in *BRCA1* and RAD51 interaction domain. The duplication causes a frameshift which changes a Valine to a Leucine at codon 1103, and creates a premature stop codon at position 6 of the new reading frame and it is predicted to cause loss of normal protein function through protein truncation and disrupt the regions required for interaction with POLH and POLH DNA synthesis stimulation, Rad51, and BRCA2, as well as the last 3 (WD5-7) WD repeats (Oliver et al., 2009; Buisson et al., 2010; Buisson et al., 2014) (PVS1 Pathogenic Very Strong). It is in a mutation hotspot and in a vicinity of 13 pathogenic variants (PM1 Pathogenic Moderate). This variant is not found in GnomAD exomes neither in GnomAD genomes (PM2 Pathogenic Moderate). 1 pathogenic prediction from GERP versus no benign prediction supports its deleterious effect (PP3 Pathogenic Supporting). The variant is reported in ClinVar as a pathogenic variant. In our study this variant was found in a 51-year-old female with a family history of cancer. Therefore, this variant is classified as a Pathogenic.

SDHB (NM_003000.2: c.269G>A; p.Arg90Gln)– This variant is in exon 3 of the *SDHB* gene in the Fer2_3 domain (F42-147Y aa); it is involved in electron transfer activity, iron-sulfur cluster binding. This missense change has been shown to reduce but not entirely abolish SDHB enzymatic activity has been reported as pathogenic in paraganglioma (Panizza et al., 2013) (PS3 Pathogenic Very Strong). This variant is in a hotspot of 10 pathogenic missense, nonsense, and frameshift variants (source ClinVar) (PM1 Pathogenic Moderate). There is a known pathogenic null (terminating) variant (i.e., c.268C>T (p.Arg90Ter) at the same amino acid residue which also suggests that this region is a mutational hotspot (PS1 Pathogenic Strong). The allele count in GnomAD exomes and GnomAD genomes are 2 and 1, respectively, which do not exceed the estimated maximal expected allele count for a pathogenic SDHB variant of 5 (PM2 Pathogenic Moderate). 11 pathogenic predictions from DANN, DEOGEN2, EIGEN, FATHMM-MKL, M-CAP, MVP, MutationAssessor, MutationTaster, PrimateAI, REVEL and SIFT versus no benign predictions support its deleterious effect (PP3 Pathogenic Supporting). This variant is reported in ClinVar as a likely pathogenic variant or a VUS. In this study this variant was found in a 46-year-old female with bilateral breast cancer and no reported family history of cancer. Based on the evidence provided above, we classified this variant as a Likely Pathogenic.

Variants of Unknown Significance (VUS)

ATM (NM_000051.3: c.7503T>A; p.Asn2501Lys)– This variant is in exon 50 of the *ATM* gene, which is involved in a double-strand break-repair pathway. The variant is in the functional domain of the *ATM* gene, called FAT domain (I1960-2566A aa) and in a mutation hotspot of 192 pathogenic variants (PM1 Pathogenic Moderate). This domain functions as a structural scaffold in an autophosphorylation site or as a protein-binding domain, possibly having a role in substrate interaction. This variant is not found in GnomAD exomes neither in GnomAD genomes (PM2 Pathogenic Moderate). 8 pathogenic predictions from DANN, FATHMM-MKL, M-CAP, MVP, MutationAssessor, MutationTaster, REVEL and SIFT versus 3 benign predictions from DEOGEN2, EIGEN and PrimateAI support its deleterious effect (PP3 Pathogenic Supporting). HGMD lists one publication for ovarian cancer where it is listed as a VUS (Kraus et al., 2017). In our study this variant was found in a 34-year-old female patient with unilateral breast cancer and a strong family history of breast cancer. Therefore, the available evidence is currently insufficient to determine its role in disease and it has been classified as a Variant of Uncertain Significance.

ATM (NM_000051.3: c.3371A>T; p.Tyr1124Phe)– This variant is in exon 18 in a non-functional domain. The Tyr1124 residue is the first codon of exon 18 just before the LZ functional domain, which plays a role in DNA damage repair. This missense variant is in a hotspot of 8 pathogenic variants (PM1 Pathogenic Moderate). The allele frequencies in GnomAD exomes is 0.000012 which does not exceed the estimated maximal expected allele frequency for a pathogenic ATM variant of 0.0001 (PM2 Pathogenic Moderate). 8 benign predictions from Align-GVGD, DANN, DEOGEN2, EIGEN, MVP, MutationTaster, PrimateAI and REVEL versus 4 pathogenic predictions from FATHMM-MKL, M-CAP, MutationAssessor support its benign effect (BP4 Benign Supporting). This variant has been observed in an individual affected with acute myeloid leukemia (Zhang, et al., 2015). This variant is reported in ClinVar as a VUS. In our study this variant was found in a 26-year-old female with unilateral breast cancer and a strong family history of cancer. The available evidence is currently insufficient to determine its role in disease. Therefore, it has been classified as a Variant of Uncertain Significance.

ATR (NM_001184.3: c.992A>G; p.Asp331Gly)– This variant was found in exon 4 of the *ATR* gene, which plays a central role in cell-cycle regulation by transducing DNA damage signals to downstream effectors of cell-cycle progression (Goodarzi, Block, & Lees-Miller, 2003). The variant is located in the ARM repeat region (E238-1369L aa) which may function as a binding site to large substrates such as proteins and nucleic acids. Only 4 missense variants in ATR gene located even far from this variant are pathogenic or likely pathogenic) (BP1 Benign Supporting). Two variants in exon 4, i.e., c.632C>T (Thr211Met) and c. 946G>A (Va131611e), of the *ATR* genes were reported in French Canadian breast and HBOC patients (Durocher et al., 2006). The allele frequency in the GnomAD exomes is 0.0014 which exceeds the estimated maximal expected allele frequency for a pathogenic ATR variant of 0.000388 derived from the 207 clinically reported variants in gene ATR (BS1 Benign Strong). Additionally, 10 benign predictions from DANN, DEOGEN2, EIGEN, FATHMM-MKL, M-CAP, MVP, MutationAssessor, MutationTaster, REVEL and SIFT versus no pathogenic predictions support its benign effect (BP4 Benign Supporting). In our study this variant was found in a 53-year-old female patient with unilateral breast cancer and a strong family history of breast cancer. Therefore, the available evidence is currently insufficient to determine its role in disease and it has been classified as a Variant of Uncertain Significance.

ATR (NM_001184.3: c.1602G>C; p.Trp534Cys)– This variant is in exon 7 of *ATR* gene in a non-

functional domain. This variant is not found in GnomAD exomes neither in GnomAD genomes (PM2 Pathogenic Moderate). 6 pathogenic predictions from DANN, EIGEN, FATHMM-MKL, MutationAssessor, MutationTaster and SIFT versus 5 benign predictions from DEOGEN2, M- CAP, MVP, PrimateAI and REVEL support its deleterious effect (PP3 Pathogenic Supporting). However, only 4 missense variants in *ATR* gene located even far from this variant are pathogenic or likely pathogenic (BP1 Benign Supporting). Although the *ATR* gene plays a significant role in DNA damage repair, there were no reports of this variant in the literature. In our study this variant was found in a 39-year-old female with unilateral breast cancer and a family history of cancer. Based on the available data, we classified this variant as a Variant of Uncertain Significance.

AXIN2 (NM_004655.3: c.2083G>T; p.Ala695Ser)– This variant is in exon 6 in a non-functional domain. The allele frequency in GnomAD exomes is 0.000164 which exceeds the estimated maximal expected allele frequency for a pathogenic AXIN2 variant of 0.0001 derived from the 1,065 clinically reported variants in gene AXIN2 (BS1 Benign Strong). This variant is also observed in healthy adults and GnomAD exomes allele count is 40 which exceeds the estimated maximal expected allele count for a pathogenic AXIN2 variant of 5 (BS2 Benign Strong). Additionally, only 1 missense variant in AXIN2 gene is known to be pathogenic (BP1 Benign Supporting). In our study this variant was found in a 28-years-old female with unilateral breast cancer and no reported family history of cancer. Thus, we classified this variant as a Variant of Uncertain Significance.

BRCA1 (NM_007294.3: c.-86C>T)– This variant is in the 5' UTR of the *BRCA1* gene. This variant has been reported in a few cases in ClinVar as a VUS. 1 benign prediction from DANN versus no pathogenic predictions supports its benign effect (BP4 Benign Supporting). In our study it was detected in a 30-year-old female patient with unilateral breast cancer and no reported family history of cancer. Therefore, due to lack of sufficient evidence we classified this variant as a Variant of Uncertain Significance.

BRCA1 (NM_007294.3: c.5191G>A; p.Glu1731Lys)– The *BRCA1* variant p.Glu1731Lys is in exon 19 in the BRCT domain (S1642-1736V aa). This domain of about 95 amino acids is found in a large variety of proteins involved in DNA repair, recombination and cell cycle control (Bork et al., 1997). It is found in a mutational hotspot including 34 pathogenic missense, nonsense, and frameshift variants (PM1 Pathogenic Moderate). The allele frequency in GnomAD exomes is 0.0000159 which does not exceed the estimated maximal expected allele frequency for a pathogenic BRCA1 variant of 0.0001, and the variant is not found in GnomAD genomes (PM2 Pathogenic Moderate). 8 pathogenic predictions from DANN, EIGEN, FATHMM-MKL, M-CAP, MVP, MutationTaster, REVEL and SIFT versus 3 benign predictions from DEOGEN2, MutationAssessor and PrimateAI support its deleterious effect (PP3 Pathogenic Supporting). In this study this variant was found in a 51-year-old female with unilateral breast cancer and a family history of cancer. Therefore, due to lack of sufficient evidence we classified this variant as a Variant of Uncertain Significance.

BRCA1 (NM_007294.3: c.5360G>A; p.Cys1787Tyr)– This variant is in exon 21 of the BRCA1 gene in the BRCT functional domain (aa.1756-1855). This domain of about 95 amino acids is found in a large variety of proteins involved in DNA repair, recombination and cell cycle control (Bork et al., 1997). The BRCT domain is not limited to the C-terminal of protein sequences and can be found in multiple copies or in a single copy as in *RAP1* and *TdT*. Some data indicated that the BRCT domain functions as a protein-protein interaction module (Zhang et al., 1998). This variant is found in a mutational hotspot of 33 pathogenic nonsense, and frameshift variants (PM1 Pathogenic Moderate). One different missense change at the same amino acid residue (chr17:41201185A>T (Cys1787Ser)) has been reported in ClinVar but with conflicting interpretations of pathogenicity. The variant is not found in GnomAD exomes neither in GnomAD genomes (PM2 Pathogenic Moderate). 11 pathogenic predictions from

DANN, EIGEN, FATHMM-MKL, M-CAP, MVP, MutationAssessor, MutationTaster, REVEL, SIFT, PolyPhen-2, and Align-GVGD versus 2 benign predictions from DEOGEN2 and PrimateAI support its deleterious effect (PP3 Pathogenic Supporting). In our study this variant was found in a 31-year-old female with unilateral breast cancer and no reported family history of cancer. Therefore, due to lack of sufficient evidence we classified this variant as a Variant of Uncertain Significance.

BRCA2 (NM_000059.3: c.8699A>T; p.Asp2900Val) and– This variant is in exon 21 of the *BRCA2* gene involved in which functions as ssDNA binding and nucleic acid recognition site; and the Tower domain (M2831-2967T aa) with a major role in tumor suppression and DNA binding. It is in a mutation hotspot with 17 pathogenic nonsense and frameshift variants (PM1 Pathogenic Moderate). The allele frequency in GnomAD exomes is 0.00000398 which does not exceed the estimated maximal expected allele frequency for a pathogenic BRCA2 variant of 0.0001, and this variant was not found in GnomAD genomes (PM2 Pathogenic Moderate). 7 pathogenic predictions from DANN, EIGEN, FATHMM-MKL, M-CAP, MutationTaster, PrimateAI and SIFT versus 2 benign predictions from MVP and REVEL support its deleterious effect (PP3 Pathogenic Supporting). One study found the p.Asp2900Tyr in a case control study of a Japanese population with breast cancer (Momozawa et al., 2018) and another study classified it as VUS in colon cancer (Yurgelun et al., 2017). This variant also is reported in ClinVar as a VUS. In our study this variant was fond in a 29-year-old female with unilateral breast cancer with family history of cancer. Therefore, due to lack of sufficient evidence we classified this variant as a Variant of Uncertain Significance.

CHEK2 (NM_007194.3: c.480A>G; p.Ile160Met)– This variant is in exon 4 of the *CHEK2* gene in the FHA functional domain (Y156-218V aa) as phosphopeptide recognition site. 13 pathogenic nonsense and frameshift variants have been reported in the vicinity of this residue Ile160, including one pathogenic variant (c.478delA (p.Ile160Terfs)) at the same position (PM1 Pathogenic Moderate). Meantime, majority of missense variants detected in CHEK2 are pathogenic and known cause of disease (PP2 Pathogenic Supporting). 8 pathogenic predictions from DANN, FATHMM-MKL, M-CAP, MVP, MutationAssessor, MutationTaster, REVEL and SIFT versus 3 benign predictions from DEOGEN2, EIGEN and PrimateAI support its deleterious effect (PP3 Pathogenic Supporting). A few studies list this variant, one study reporting partial loss of function for *CHEK2* due to this variant (Roeb et al., 2012), and two others finding it in breast cancer patients (Mohamad, 2015). This variant is also reported in ClinVar as a VUS. In our study it was found in a 36-year-old female with unilateral breast cancer and family history of breast cancer. Based on the evidence above, we classified this variant as a Variant of Uncertain Significance.

CHEK2 (NM_007194.4: c.1312G>T; p.Asp438Tyr)– This variant is in exon 12 of the *CHEK2* gene's Pkinase domain (M265-529L aa); this domain contains the catalytic function of protein kinases. One study described that this variant reduced kinase activity by 70% (Bell et al., 2007), however, it is unclear whether such a reduction in kinase activity would be sufficient to contribute to cancer risk. It was reported to be associated with prostate cancer (Southey et al., 2016), and as VUS in male breast cancer (Godar & Guy, 2010). It was also reported in colorectal cancer (Kayser et al., 2018). It is in a hotspot of 7 pathogenic nonsense and frameshift variants (PM1 Pathogenic Moderate). Although majority of missense variants detected in CHEK2 are pathogenic (PP2 Pathogenic Supporting), this variant which is also reported as p.Asp481Tyr and has been reported in several studies in individuals with a history of breast or prostate cancer, as well as in healthy controls. In our study this variant was found in two sisters with breast cancer at age 44 and 51 and family history of breast cancer. Based on currently available evidence, we consider it to be as a Variant of Uncertain Significance.

FANCB (NM_001018113.1: c.1480A>G; p.Thr494Ala)– This variant is in exon 7 of the *FANCB* gene in a non-functional domain (M7-858aa), which could mediate *FANCD2* ubiquitination and plays a role in FA DNA damage response pathway and germ cell development. This variant is not found in GnomAD exomes neither in GnomAD genomes (PM2 Pathogenic Moderate). Only 3 out of 41 non-VUS missense variants in FANCB gene are pathogenic or likely pathogenic (BP1 Benign Supporting). 5 benign predictions from DEOGEN2, M-CAP, MVP, MutationTaster and REVEL versus 4 pathogenic predictions from DANN, FATHMM-MKL, MutationAssessor and SIFT support its benign effect (BP4 Benign Supporting). In our study this variant was found in a 36-year-old female with unilateral breast cancer and no reported family history. Therefore, the available evidence is currently insufficient to determine its role in disease, and it has been classified as a Variant of Uncertain Significance.

FANCD2 (NM_033084.4: c.1777C>T; p.Pro593Ser)– This variant is in exon 20 of the *FANCD2* gene in the *FANCD2* domain (P90-1403N aa), where monoubiquitinated *FANCD2* functions to recruit DNA repair factors. The allele frequency in GnomAD exomes is 0.000883 which exceeds the estimated maximal expected allele frequency for a pathogenic FANCD2 variant of 0.000584 derived from the 210 clinically reported variants in gene FANCD2 (BS2 Benign Strong). Only 4 out of 34 non-VUS missense variants in FANCD2 gene are pathogenic or likely pathogenic (BP1 Benign Supporting). 11 benign predictions from DANN, DEOGEN2, EIGEN, FATHMM-MKL, M-CAP, MVP, MutationAssessor, MutationTaster, PrimateAI, REVEL and SIFT versus no pathogenic predictions support its benign effect (BP4 Benign Supporting). The variant is reported in ClinVar as a VUS. In our study this variant was found in a 33-year-old female with unilateral breast cancer and no reported family history of cancer. Therefore, the available evidence is currently insufficient to determine its role in disease, and it has been classified as a Variant of Uncertain Significance.

FANCI (NM_001113378.1: c.3812C>T; p.Ser1271Phe)– This variant is in exon 36 of the *FANCI* gene in the *FANCI*_S4 domain (L1046 -1294L aa), which is thought to play a role in DNA repair. The allele frequency in GnomAD exomes is 0.0000716 which does not exceed the estimated maximal expected allele frequency for a pathogenic FANCI variant of 0.0001, and the variant is not found in GnomAD genomes (PM2 Pathogenic Moderate). 30 of 32 non-VUS missense variants in FANCI gene are benign (BP1 Benign Supporting). 10 pathogenic predictions from DANN, EIGEN, FATHMM-MKL, M-CAP, MVP, MutationAssessor, MutationTaster, PrimateAI, REVEL and SIFT versus 1 benign prediction from DEOGEN2 support its deleterious effect (PP3 Pathogenic Supporting). In our study this variant was found in a 34-year-old female with unilateral breast cancer with a family history of cancer. Based on the findings above, we consider this variant as a Variant of Uncertain Significance.

MC1R (NM_002386.3: c.104G>A; p.Cys35Tyr)– This variant is in exon 1 of the *MC1R* gene one residue before a structural domain (S36-49G aa) begins. This variant was observed in healthy adults, and the allele count in GnomAD exomes is 21 which exceeds the estimated maximal expected allele count for a pathogenic MC1R variant of 5 (BP1 Benign Supporting). 30 of 32 non-VUS missense variants in FANCI gene are benign (BP1 Benign Supporting). This variant has been reported in cases with melanoma as a VUS (Hu et al., 2014; Ibarrola-Villava et al., 2014; Kanetsky et al., 2006; Landi et al., 2006). The *MC1R* gene plays an indirect role in melanoma yet no distinct association of the mutations in this gene and other cancer has been established. 6 pathogenic predictions from EIGEN, FATHMM-MKL, M-CAP, MutationAssessor, MutationTaster and SIFT versus 4 benign predictions from DANN, DEOGEN2, MVP and REVEL support its deleterious effect (PP3 Pathogenic Supporting). In this study this variant was found in a 43-year-old female with bilateral breast cancer and no reported family history. Based on the findings above, we consider this variant as a Variant of Uncertain Significance.

MLH1 (NM_000249.3: c.954C>A; p.His318Gln)– This variant is in exon 11 of the *MLH1* gene, in the functional DNA Mismatch Repair domain (I216-334L aa), which is an ATP binding site. This is a mutation hotspot of 22 pathogenic nonsense and frameshift variants (PM1 Pathogenic Moderate). This variant is not found in GnomAD exomes neither in GnomAD genomes (PM2 Pathogenic Moderate). 207 out of 273 non-VUS missense variants in gene MLH1 are pathogenic (PP2 Pathogenic Supporting). 6 benign predictions from DANN, EIGEN, FATHMM-MKL, MVP, PrimateAI and REVEL versus 5 pathogenic predictions from DEOGEN2, M-CAP, MutationAssessor, MutationTaster and SIFT **do not agree on the potential impact of this missense change**. The variant is reported in ClinVar as a VUS. This variant has not been reported in the literature in individuals with MLH1-related disease. In this study this variant was found in a 49-year-old female with unilateral breast cancer and a strong family history of cancer. Based on the evidence above, we classified this variant as a Variant of Uncertain Significance.

MSH6 (NM_000179.2: c.3727A>T; p.Thr1243Ser)– This variant is in exon 8 of *MSH6*, a mismatch repair gene, in domain mutATP5 (V1127-1321R aa), which functions in ATPase activity. It is in a mutation hotspot of 19 pathogenic nonsense and frameshift variants (PM1 Pathogenic Moderate). The allele frequency in South Asians in GnomAD exomes is 0.00114 which exceeds the estimated maximal expected allele frequency for a pathogenic MSH6 variant of 0.000649 derived from the 4,260 clinically reported variants (BS1 Benign Strong). 7 pathogenic predictions from DEOGEN2, FATHMM-MKL, M-CAP, MVP, MutationTaster, PrimateAI and SIFT versus 4 benign predictions from DANN, EIGEN, MutationAssessor and REVEL support its deleterious effect (PP3 Pathogenic Supporting). It has been reported in a study in mesothelioma as a VUS and in ovarian cancer (Pal et al., 2012; Betti et al., 2017). In our study this variant was found in a 51-year-old female with bilateral breast cancer and no reported family history. Based on the findings above, we consider this variant as a Variant of Uncertain Significance.

MUTYH (NM_001128425.1: c.553C>T; p.Arg185Trp)– The *MUTYH* gene variant p.Arg185Trp is in exon 7 and in the HhH-GPD domain (V118-249W aa) with a function in base-excision repair (Bruner, Norman, & Verdine, 2000). Experimental studies showed this missense change abolishes the glycosylase activity of the MUTYH protein (D'Agostino et al., 2010; Molatore et al., 2010) (PS3 Pathogenic Strong). It is in a mutation hotspot of 7 pathogenic, including missense pathogenic variants (PM1 Pathogenic Moderate). The allele frequency in GnomAD exomes is 0.00000795 which does not exceed the estimated maximal expected allele frequency for a pathogenic MUTYH variant of 0.0001, and the variant is not found in GnomAD genomes (PM2 Pathogenic Moderate). Meantime, majority of missense variants detected in MUTYH are pathogenic and known cause of disease (PP2 Pathogenic Supporting). In our study this variant was found in a 54-year-old female patient with unilateral breast cancer and a family history of cancer. Therefore, the available evidence is currently insufficient to determine its role in disease, and it has been classified as a Variant of Uncertain Significance.

MUTYH (NM_001128425.1: c.1258C>A; p.Leu420Met)– This variant is in exon 13 of the *MUTYH* gene, involved in tumor suppression through apoptosis, in domain DNA_Glycosylase_C (R354-483Y aa), which is involved in Adenine DNA glycosylation. This is in a mutation hotspot of 12 pathogenic variants (PM1 Pathogenic Moderate). Despite only a few pathogenic variants have been reported upstream of this residue with no indication that this region is a mutational hotspot, 61 of 104 non-VUS missense variants in gene MUTYH are pathogenic (PP2 Pathogenic Supporting). **The variant was observed in the large, broad control population, ExAC, with an allele frequency of 71/120676 (1/1699), which does not exceed the estimated maximal expected allele frequency for a pathogenic MUTYH variant of 1/219 (PM2 Pathogenic Moderate).** 7 pathogenic predictions from DANN, FATHMM-MKL, M-CAP, MVP, MutationAssessor, MutationTaster and SIFT versus 4 benign predictions from DEOGEN2, EIGEN, PrimateAI and REVEL support its deleterious effect (PP3 Pathogenic

Supporting). This variant has been reported in a family with HNPCC, breast, pancreatic, and colorectal cancers, and its functional analysis indicates the variant to act comparable to wild type function (Komine et al., 2015). Meantime, MUTYH heterozygous or homozygous mutations among breast cancer patients with or without history of the disease evidenced an association of MUTYH with an increased risk of BC (Rennert et al., 2012; Dutil et al., 2019; Cuchra et al., 2016; Win et al., 2016). In our study this variant was found in two unrelated patients: a 55-year-old female with bilateral breast cancer and a 40-year-old female with ovarian and breast cancer and both with family history of cancer. Therefore, the available evidence is currently insufficient to determine its role in disease, and it has been classified as a Variant of Uncertain Significance.

PALB2 (NM_024675.3: c.833_834delTAinsAT; p.Leu278His)– This variant is in a non- functional domain (200- 394 aa), between DNA binding and CHAM functional domains. However, this is in a mutation of hotspot of 12 pathogenic nonsense and frameshift variants (PM1 Pathogenic Moderate). This variant is not found in GnomAD exomes neither in GnomAD genomes (PM2 Pathogenic Moderate). 1 benign prediction from GERP versus no pathogenic predictions supports its deleterious effect (PP3 Pathogenic Supporting). This variant is reported in ClinVar as a VUS. This variant has not been reported in the literature in individuals with PALB2-related disease. In this study this variant was found in a 41-year-old female with unilateral breast cancer and a strong family history of breast cancer. Based on this evidence, we consider this variant as a Variant of Uncertain Significance.

PALB2 (NM_024675.3: c.2821A>G; p.Ile941Val)– This variant is in exon 8 of the *PALB2* gene, which is involved in repairing double strand DNA breaks, in the WD40 repeat-like (V872-1185 aa) domain, which serves as platform for the assembly of protein complexes (e.g., *BRCA1* / RAD51) or mediators of transient interplay among other proteins. It is in a mutation hotspot with 150 pathogenic variants (PM1 Pathogenic Moderate). The allele frequency in GnomAD exomes is 0.0000199 which does not exceed the estimated maximal expected allele frequency for a pathogenic PALB2 variant of 0.0001, and this variant is not found in GnomAD genomes (PM2 Pathogenic Moderate). 8 pathogenic predictions from DANN, EIGEN, FATHMM-MKL, MutationAssessor, MutationTaster, SIFT, PolyPhen-2, Align-GVGD versus 5 benign predictions from DEOGEN2, M-CAP, MVP, PrimateAI and REVEL support its deleterious effect (PP3 Pathogenic Supporting). This variant is reported in ClinVar as a VUS. In this study this variant was found in a 55-year-old female with bilateral breast cancer and a family history of breast cancer. Based on this evidence, we consider this variant as a Variant of Uncertain Significance.

PALB2 (NM_024675.3: c.3428T>A; p.Leu1143His)– This variant is in exon 8 of the *PALB2* gene, which is involved in repairing double-strand DNA breaks in WD40 repeat-like (V872- 1185 aa) domain, which serves as platforms for the assembly of protein complexes (e.g., *BRCA1* / RAD51) or mediators of transient interplay among other proteins. This variant is in a hotspot of 12 pathogenic nonsense and frameshift variants, including one mutation (i.e., c.3426dupA; p.Leu1143Thrfs) at the same position (Source ClinVar) (PM1 Pathogenic Moderate). It has been reported in several cancers including breast cancers (Yadav, Reeves, Campian, Paine, & Zakalik, 2017; Grant et al., 2015; Ramus et al., 2015; Thompson et al., 2015; Hellebrand et al., 2011). Furthermore, it has been experimentally shown that this missense change modestly reduces DNA double-stranded break-induced homologous recombination, affects the PALB2 protein interaction with RAD51C, XRCC3 and BRCA2 proteins and moderately increases cellular sensitivity to ionizing radiation (Park et al., 2014). 6 pathogenic predictions from EIGEN, FATHMM-MKL, M-CAP, MutationAssessor, MutationTaster and SIFT vs 5 benign predictions from DANN, DEOGEN2, MVP, PrimateAI and REVEL support its deleterious effect (PP3 Pathogenic Supporting). This variant was found in a 46-years old female with bilateral breast cancer and no reported family history of cancer. In summary, the available evidence is currently insufficient to determine the

role of this variant in the disease. Therefore, it has been classified as a Variant of Uncertain Significance.

SLX4 (NM_032444.3: c.421G>T; p.Gly141Trp)– This variant is in a structural domain of the *SLX4* gene in exon 2. The allele frequency in GnomAD exomes is 0.000819 which exceeds the estimated maximal expected allele frequency for a pathogenic SLX4 variant of 0.000165 derived from the 559 clinically reported variants in gene SLX4 (BS1 Benign Strong). 9 benign predictions from DANN, DEOGEN2, EIGEN, FATHMM-MKL, MVP, MutationAssessor, MutationTaster, PrimateAI and REVEL versus 4 pathogenic predictions from M-CAP, SIFT, PolyPhen-2, Align-GVGD support its benign effect (BP4 Benign Supporting). This variant is reported in ClinVar as a VUS. This variant has been reported in individuals affected with breast cancer, childhood acute lymphoblastic leukemia, and unspecified cancer types (De Garibay et al., 2013; Shah et al., 2013; Fernández-Rodríguez et al., 2012; Jalkh et al., 2017; Spinella et al., 2015; Cabanillas et al., 2017). In our study it was found in a 29-year-old female with unilateral breast cancer and a family history of cancer. Therefore, the available evidence is currently insufficient to determine the role of this variant in disease and this variant has been classified as a Variant of Uncertain Significance.

SLX4 (NM_032444.2: c.2320G>T; p.Ala774Ser)– This variant was found in exon 11 of the *SLX4* gene in the BTB domain (M684-789V aa), functioning as transcriptional regulators with TRF2 and RAP1 genes. This variant is not found in GnomAD genomes (PM2 Pathogenic Moderate). 8 pathogenic predictions from DANN, DEOGEN2, EIGEN, FATHMM-MKL, M-CAP, MutationAssessor, MutationTaster and SIFT versus 3 benign predictions from MVP, PrimateAI and REVEL support its deleterious effect (PP3 Pathogenic Supporting). In our study it was found in two unrelated patients– a 27-year-old female with unilateral breast cancer and no reported family history of cancer and a 28-year-old female with unilateral breast cancer and a strong family history of cancer. However, the available evidence is currently insufficient to determine the role of this variant in disease and this variant has been classified as a Variant of Uncertain Significance.

SLX4 (NM_032444.2: c.4423A>G; p.Thr1475Ala)– This variant is in exon 12 of the *SLX4* gene in a non-functional domain before the functional SBD domain (E1754- 1810T aa). This variant is not found in GnomAD exomes neither in GnomAD genomes (PM2 Pathogenic Moderate). 9 benign predictions from DANN, DEOGEN2, EIGEN, MVP, MutationAssessor, MutationTaster, PrimateAI, REVEL and SIFT versus 2 pathogenic predictions from FATHMM-MKL and M-CAP support its deleterious effect (PP3 Pathogenic Supporting). In our study this variant was found in a 35-year-old female with unilateral breast cancer and no reported family history. Based on the limited evidence below and the fact that this variant has not been reported before in disease, we consider this variant as a Variant of Uncertain Significance.

SLX4 (NM_032444.2: c.5413T>C; p.Cys1805Arg)– The missense variant p.Cys1805Arg is in exon 15 of the *SLX4* gene in the structure-specific endonuclease subunit *SLX4* domain (D1753-1810T aa). *SLX4* is a regulatory subunit of the *SLX1-SLX4* structure-specific endonuclease that resolves DNA secondary structures generated during DNA repair and recombination (Muñoz et al., 2009; Fekairi et al., 2009). The allele frequency in GnomAD exomes is 0.000008 which does not exceed the estimated maximal expected allele frequency for a pathogenic SLX4 variant of 0.0001, and the variant is not found in GnomAD genomes (PM2 Pathogenic Moderate). 6 pathogenic predictions from DEOGEN2, FATHMM-MKL, M-CAP, MutationAssessor, MutationTaster and SIFT vs 5 benign predictions from DANN, EIGEN, MVP, PrimateAI and REVEL support its deleterious effect (PP3 Pathogenic Supporting). In our study this variant was found in two unrelated female patients with unilateral breast cancer– a 45-year-old patient with a strong family history of breast cancer and a 26-year-old patient with no reported family history of cancer. Based on the limited evidence and the fact that this variant has not been reported before in disease,

we consider this variant as a Variant of Uncertain Significance.

SMARCA4 (NM_001128849.1: c.403C>G; p.Pro135Ala)– This variant is in exon 4 of the *SMARCA4* gene in a non-functional domain at the N terminal of the protein just outside the QLQ domain (T170-206G aa), a protein interaction domain. The allele frequency in GnomAD exomes is 0.000193 which exceeds the estimated maximal expected allele frequency for a pathogenic SMARCA4 variant of 0.0001 derived from the 1,851 clinically reported variants (BS1 Benign Strong). This is also observed in healthy adults: the allele count in GnomAD exomes is 48 which exceeds the estimated maximal expected allele count for a pathogenic SMARCA4 variant of 5 (BS1 Benign Strong). 7 benign predictions from DANN, DEOGEN2, EIGEN, MVP, MutationAssessor, PrimateAI and REVEL versus 4 pathogenic predictions from FATHMM-MKL, M-CAP, MutationTaster and SIFT support its benign effect (BP4 Benign Supporting). This variant is reported in ClinVar as a likely benign. In our study this variant was found in a 40-year-old female with bilateral breast cancer and a family history of cancer. Therefore, the available evidence is currently insufficient to determine the role of this variant in disease and this variant has been classified as a Variant of Uncertain Significance.

TSHR (NM_000369.2: c.202C>T; p.Pro68Ser)– This variant is in exon 2 of the *TSHR* gene, in a functional LRR_5 domain (I60-125L aa), which is involved in the formation of protein-protein interactions. 39 of 53 non-VUS missense variants in gene TSHR are pathogenic (PP2 Pathogenic Supporting). Surface expression of the p.Pro68Ser variant was found to be reduced by 80% in HEK-293 cells and by 50% in COS-7 cells compared to wild type, but the biological activity as measured by cAMP production was not affected (Tenenbaum-Rakover et al., 2009) (PS3 Pathogenic Strong). 9 pathogenic predictions from DANN, DEOGEN2, EIGEN, FATHMM-MKL, M-CAP, MVP, MutationAssessor, MutationTaster and REVEL versus 2 benign predictions from PrimateAI and SIFT support its deleterious effect (PP3 Pathogenic Supporting). This variant has been reported as pathogenic or likely pathogenic in thyroid gland disorders (Tenenbaum-Rakover et al., 2015) and cancers such as Seminoma and Merkel cell carcinoma (Schrader et al., 2016). In our study it was found in a 28-year-old female with unilateral breast cancer and no reported family history of cancer. Although this variant has been seen in cancers, its role in breast cancer is unknown; thus, based on the insufficient evidence above, this variant has been classified as a Variant of Uncertain Significance.

WRAP53 (NM_018081.2: c.838G>A; p.Ala280Thr)– This variant is in exon 6 of the *WRAP53* gene in the functional WD40 domain (H281-303F aa), encoding RNA antisense to TP53. It also acts as a site for protein-protein interaction and is known to serve as platform for the assembly of protein complexes or mediators of transient interplay among other proteins. The allele frequency in GnomAD exomes is 0.0000119 which does not exceed the estimated maximal expected allele frequency for a pathogenic WRAP53 variant of 0.0001, and this variant is not found in GnomAD genomes (PM2 Pathogenic Moderate). 6 pathogenic predictions from DANN, EIGEN, FATHMM-MKL, M-CAP, MutationTaster and SIFT versus 4 benign predictions from DEOGEN2, MVP, PrimateAI and REVEL support its deleterious effect (PP3 Pathogenic Supporting). However, 13 of 14 of non-VUS missense variants in gene WRAP53 are benign (BP1 Benign Supporting). Other variants in the *WRAP53* gene have been reported in breast and ovarian cancers (Medrek et al., 2013). In our study this variant was found in a 31-year-old female with unilateral breast cancer and no reported family history. Based on the limited evidence below and the fact that this variant has not been reported before in disease, we consider this variant as a Variant of Uncertain Significance.

WRAP53 (NM_018081.2: c.1564dup; p.Ala522Argfs)– This variant is in exon 10 of the *WRAP53* gene in a structural domain just before the glycine-rich domain. The truncation of this protein due to this frameshift variant occurs in the C terminus of the protein, and most of the cancer related variants have

been reported before this frameshift variant. The allele frequency in East Asians in GnomAD exomes is 0.0012 which exceeds the estimated maximal expected allele frequency for a pathogenic WRAP53 variant of 0.000498 derived from the 61 clinically reported variants in gene WRAP53 (BS1 Benign Strong). 1 pathogenic prediction from GERP versus no benign predictions supports its deleterious effect (PP3 Pathogenic Supporting). In our study this variant was found in a female patient with unilateral breast cancer at age 27 with no reported family history of cancer. It was also found in another female patient at age 28 with a strong family history of cancer, yet this patient also had a *SLX4* variant (i.e., (c.2320G>T; p.Ala774Ser), which was classified as a VUS. Based on the limited evidence and the fact that this variant has not been reported before in cancer, we consider this variant as a Variant of Uncertain Significance.

XRCC2 (NM_005431.1: c.268C>T; p.Leu90Phe)– This variant is in exon 3 in the RECA-like ATP-binding functional domain (H60-248Q aa), which is part of the RAD51 complex in homologous recombination repair (IPR013632). The core ATP-binding site domain is well conserved with 14 invariant residues. It contains the nucleotide binding loop between beta- strand 1 and alpha-helix C. It interacts selectively and non-covalently with ATP, adenosine 5'- triphosphate, a universally important coenzyme and enzyme regulator (source: InterPro ebi.ac.uk/interpro/protein/O43543). This variant is not found in GnomAD exomes neither in GnomAD genomes (PM2 Pathogenic Moderate). However, all 8 non-VUS missense variants in gene XRCC2 are benign (BP1 Benign Supporting). Additionally, 6 benign predictions from DEOGEN2, EIGEN, M-CAP, MVP, PrimateAI and REVEL versus 5 pathogenic predictions from DANN, FATHMM-MKL, MutationAssessor, MutationTaster and SIFT support its benign effect (BP4 Benign Supporting). In our study this variant was found in a 26-year-old female with unilateral breast cancer and a strong family history of cancer. This variant has not been reported in breast cancer patients before. In summary, the available evidence is currently insufficient to determine the role of this variant in disease. Therefore, it has been classified as a Variant of Uncertain Significance.

References

Agrawal V., & Kishan K.V. (2003). OB-fold: growing bigger with functional consistency. Current Protein and Peptide Science, 4, 195-206.

Antoniou A., et al. (2014). Breast-cancer Risk in Families With Mutations in PALB2. N Engl J Med. 371(6):497-506.

Barletta J.A., & Hornick J.L. (2012). Succinate dehydrogenase-deficient tumors: diagnostic advances and clinical implications. Advances in Anatomic Pathology, 19(4), 193-203.

Beckta J.M., et al. (2015). Mutation of the BRCA1 SQ-cluster results in aberrant mitosis, reduced homologous recombination, and a compensatory increase in non-homologous end joining. Oncotarget, 6, 27674-27687.

Bell D.W., et al. (2007). Genetic and functional analysis of CHEK2 (CHK2) variants in multiethnic cohorts. International Journal of Cancer, 121, 2661-2667.

Betti M., et al. (2017). Germline mutations in DNA repair genes predispose asbestos-exposed patients to malignant pleural mesothelioma. Cancer Letters, 405, 38-45.

Borg A., et al. (2010). Characterization of BRCA1 and BRCA2 Deleterious Mutations and Variants of Unknown Clinical Significance in Unilateral and Bilateral Breast Cancer: The WECARE Study. Human Mutations, 31(3), E1200-E1240.

Bork P., et al. (1996). Internal repeats in the BRCA2 protein sequence. National Genetics, 13, 22-23.

Bork P., et al. (1997). A superfamily of conserved domains in DNA damage-responsive cell cycle checkpoint proteins. FASEB Journal, 11, 68-76.

Bruner S.D., et al. (2000). Structural basis for recognition and repair of the endogenous mutagen 8-oxoguanine in DNA. Nature, 403, 859-866.

Cabanillas R., et al. (2017). A Novel Molecular Diagnostics Platform for Somatic and Germline Precision Oncology. Mol Genet Genomic Med. 5(4):336-359.

Cardaci S., & Ciriolo M.R. (2012). TCA Cycle Defects and Cancer: When Metabolism Tunes Redox State. International Journal of Cell Biology, 2012, 161837.

Chen P.L., et al. (1998). The BRC repeats in BRCA2 are critical for RAD51 binding and resistance to methyl methanesulfonate treatment. Proceedings of the National Academy of Sciences of the USA, 95, 5287-5292.

Cuchra M., et al. (2016). The role of base excision repair in pathogenesis of breast cancer in the Polish population. Mol Carcinog. 2016;55(12):1899-1914.

D'Agostino VG, et al. (2010). Functional Analysis of MUTYH Mutated Proteins Associated With Familial Adenomatous Polyposis. DNA Repair (Amst). 9(6):700-7.

De Garibay G.R., et al. (2013). Low prevalence of SLX4 loss-of-function mutations in non-BRCA1/2 breast and/or ovarian cancer families. European Journal of Human Genetics, 21, 883-886.

Dillenburg C.V, et al. (2012). Prevalence of 185delAG and 5382insC mutations in BRCA1, and 6174delT in BRCA2 in women of Ashkenazi Jewish origin in southern Brazil. Genetics and Molecular Biology, 35(3), 599–602.

Durocher F., et al. (2006). Mutation analysis and characterization of ATR sequence variants in breast cancer cases from high-risk French-Canadian breast/ovarian cancer families. BMC Cancer, 6, 230.

Dutil J, et al. (2019). Germline variants in cancer genes in high-risk non-BRCA patients from Puerto Rico. Sci Rep. 9(1):17769.

Falck J., et al. (2001). Functional Impact of Concomitant Versus Alternative Defects in the Chk2-p53 Tumour Suppressor Pathway. Oncogene. 20(39):5503-10.

Fekairi S., et al. (2009). Human SLX4 is a Holliday junction resolvase subunit that binds multiple DNA repair/recombination endonucleases. Cell, 138, 78-79.

Fernández-Rodríguez J., et al. (2012). Analysis of SLX4/FANCP in non-BRCA1/2-mutated Breast Cancer Families. BMC Cancer. 8;12:84.

P.J., et al. (2018). VariantValidator: Accurate validation, mapping and formatting of sequence variation descriptions. Human Mutation.39, 61–68.

Gamsjaeger R., et al. (2007). Sticky fingers: zinc-fingers as protein-recognition motifs. Trends in Biochemical Sciences, 32, 63-70.

Godar S., & Guy H. (2010). Managing highly exuding wounds with Eclypse dressings. British Journal of Nursing, 19, S26-29.

Goodarzi A.A., et al. (2003). The role of ATM and ATR in DNA damage-induced cell cycle control. Prog Cell Cycle, 5, 393-411.

Grant R.C., et al. (2015). Prevalence of germline mutations in cancer predisposition genes in patients with pancreatic cancer. Gastroenterology, 148, 556-564.

Guidugli L., et al. (2018). Assessment of the Clinical Relevance of BRCA2 Missense Variants by Functional and Computational Approaches. American Journal of Human Genetics, 102, 233-248.

Hellebrand H., et al. (2011). Germline mutations in the PALB2 gene are population specific and occur with low frequencies in familial breast cancer. Hum Mutat. 32(6): E2176-88.

Hu H.H., et al. (2014). A Large French Case-Control Study Emphasizes the Role of Rare Mc1R Variants in Melanoma Risk. Biomed Res Int. 2925716.

Jalkh N., et al. (2017). Next-generation Sequencing in Familial Breast Cancer Patients From Lebanon. BMC Med Genomics. 10(1):8.

Ibarrola-Villava M., et al. (2014). Modeling MC1R Rare Variants: A Structural Evaluation of Variants Detected in a Mediterranean Case-Control Study. J Invest Dermatol. 134(4):1146-1149.

Janatova M., et al. (2013). The PALB2 Gene Is a Strong Candidate for Clinical Testing in BRCA1- And BRCA2-negative Hereditary Breast Cancer. Cancer Epidemiol Biomarkers Prev. 22(12):2323-32.

Kanetsky P.A., et al. (2006). Population-based Study of Natural Variation in the melanocortin-1 Receptor Gene and Melanoma. Cancer Res. 66(18):9330-7.

Kayser K., et al. (2018). Copy number variation analysis and targeted NGS in 77 families with suspected Lynch syndrome reveals novel potential causative genes. International Journal of Cancer, 143, 2800-2813.

Kilpivaara O., et al. (2004). CHEK2 Variant I157T May Be Associated With Increased Breast Cancer Risk. Int J Cancer. 111(4):543-7.

Kim Y., et al. (2011). Mutations of the SLX4 Gene in Fanconi Anemia. Nature Genetics, 43, 142-146.

Komine K., et al. (2015). Functional Complementation Assay for 47 MUTYH Variants in a MutY-Disrupted Escherichia coli Strain. Human Mutation, 36, 704-711.

Kraus C., et al. (2017). Gene panel sequencing in familial breast/ovarian cancer patients identifies multiple novel mutations also in genes others than BRCA1/2. International Journal of Cancer, 140,95-102.

LaDuca H., et al. (2014). Utilization of multigene panels in hereditary cancer predisposition testing: Analysis of more than 2,000 patients. Genetics in Medicine, 43(4), 295-305.

Landi MT, et al. (2006). MC1R Germline Variants Confer Risk for BRAF-mutant Melanoma. Science. 313(5786):521-2.

Li J., et al. (2002). Structural and Functional Versatility of the FHA Domain in DNA-damage Signaling by the Tumor Suppressor Kinase Chk2. Mol Cell. 9(5):1045-54.

Mahmoudi, S., et al. (2011). WRAP53 promotes cancer cell survival and is a potential target for cancer therapy. Cell Death & Disease, 2, e114.

Maxwell K.N., et al. (2016). Evaluation of ACMG-Guideline-Based Variant Classification of Cancer Susceptibility and Non-Cancer-Associated Genes in Families Affected by Breast Cancer. American Journal of Human Genetics, 98, 801-817.

Medrek K., et.al. (2013). Association of common WRAP 53 variant with ovarian cancer risk in the Polish population. Molecular Biology Reports 40, 2145-2147.

Mohamad S., et.al. (2015). Low Prevalence of CHEK2 Gene Mutations in Multiethnic Cohorts of Breast Cancer Patients in Malaysia. PLoS One. 28;10(1): e0117104.

Molatore S., et al. (2010). MUTYH Mutations Associated With Familial Adenomatous Polyposis: Functional Characterization by a Mammalian Cell-Based Assay. Hum Mutat. 31(2):159-66.

Momozawa Y., et al. (2018). Germline pathogenic variants of 11 breast cancer genes in 7,051 Japanese patients and 11,241 controls. Nature Communications, 9, 4038.

Muñoz I.M., et al. (2009). Coordination of structure-specific nucleases by human SLX4/BTBD12 is required for DNA repair. Molecular Cell, 35, 116-127.

Panizza E., et al. (2013). Yeast model for evaluating the pathogenic significance of SDHB, SDHC and SDHD mutations in PHEO-PGL syndrome. Human Molecular Genetics, 22, 804-815.

Park J.Y., et al. (2014). Breast cancer-associated missense mutants of the PALB2 WD40 domain, which directly binds RAD51C, RAD51 and BRCA2, disrupt DNA repair. Oncogene 33, 4803-4812.

Prakash S., & Prakash L. (2000). Nucleotide excision repair in yeast. Mutation Research, 451, 13-24.

Rahman N., et al. (2007). PALB2, Which Encodes a BRCA2-interacting Protein, Is a Breast Cancer Susceptibility Gene. Nat Genet. 39(2):165-7.

Ramus S.J., et al. (2015). Germline Mutations in the BRIP1, BARD1, PALB2, and NBN Genes in Women With Ovarian Cancer. Journal of the National Cancer Institute, 107, pii.djv214.

Ready K.J., et al. (2009). Accuracy of the BRCAPRO model among women with bilateral breast cancer. Cancer, 115, 725–730.

Rennert G., et al. (2012). MUTYH mutation carriers have increased breast cancer risk. Cancer. 118(8):1989-93.

Richards S., et al. (2015). Standards and guidelines for the interpretation of sequence variants: a joint consensus recommendation of the American College of Medical Genetics and Genomics and the Association for Molecular Pathology. Genetics in Medicine, 17(5), 405-424.

Roeb W., et al. (2012). Response to DNA damage of CHEK2 missense mutations in familial breast cancer. Human Molecular Genetics, 21, 2738-2744.

Santos C., et al. (2009). Haplotype and Quantitative Transcript Analyses of Portuguese Breast/Ovarian Cancer Families With the BRCA1 R71G Founder Mutation of Galician Origin. Fam Cancer. 8(3):203-8.

Schrader K.A., et al. (2016). Germline Variants in Targeted Tumor Sequencing Using Matched Normal DNA. Journal of the American Medical Association Oncology, 2, 104-111.

Shah S., et al. (2013). Assessment of SLX4 Mutations in Hereditary Breast Cancers. PLoS One, 8, e66961.

Southey M.C., et al. (2016). PALB2, CHEK2 and ATM rare variants and cancer risk: data from COGS. Journal of Medical Genetics, 53, 800-811.

Spinella JF, et al. (2015). Whole-exome Sequencing of a Rare Case of Familial Childhood Acute Lymphoblastic Leukemia Reveals Putative Predisposing Mutations in Fanconi Anemia Genes. BMC Cancer. 15:539.

Stone E.A, & Sidow A. (2005). Physicochemical constraint violation by missense substitutions mediates impairment of protein function and disease severity. Genome Research, 15, 978-986.

Sun C.K., et al. (2014). TCAB1: a potential target for diagnosis and therapy of head and neck carcinomas. Molecular Cancer, 13, 180.

Tavtigian S.V., et al. (2006). Comprehensive statistical study of 452 BRCA1 missense substitutions with classification of eight recurrent substitutions as neutral. Journal of Medical Genetics, 3, 295–305.

Tenenbaum-Rakover Y., et al. (2015). Long-term outcome of loss-of-function mutations in thyrotropin receptor gene. Thyroid, 25, 292-299.

Thompson E.R., et al. (2015). Prevalence of PALB2 mutations in Australian familial breast cancer cases and controls. Breast Cancer Research, 17, 111.

Tung N., et al. (2016). Frequency of Germline Mutations in 25 Cancer Susceptibility Genes in a Sequential Series of Patients With Breast Cancer. Journal of Clinical Oncology, 34, 1460-1468.

Vega A., et al. (2001). The R71G BRCA1 Is a Founder Spanish Mutation and Leads to Aberrant Splicing of the Transcript. Hum Mutat. 17(6):520-1.

Wilson J.S., et al. (2013). Localization-dependent and independent roles of SLX4 in regulating telomeres. Cell Reports, 4(5), 853-60.

Win A.K., et al. (2016). Risk of extracolonic cancers for people with biallelic and monoallelic mutations in MUTYH. Int J Cancer. 139(7):1557-63.

Xu X., et al. (1999). Centrosome amplification and a defective G2-M cell cycle checkpoint induce genetic instability in BRCA1 exon 11 isoform-deficient cells. Molecular Cell, 3, 389-395.

Yadav S., et al. (2017). Outcomes of retesting BRCA negative patients using multigene panels. Familial Cancer, 16, 319-328.

Yang H., et al. (2002). BRCA2 function in DNA binding and recombination from a BRCA2-DSS1-ssDNA structure. Science, 297, 1837-1848.

You Z., et al. (2005). ATM activation and its recruitment to damaged DNA require binding to the C terminus of Nbs1. Molecular Cell Biology, 25, 5363-5379.

Yurgelun M.B, et al. (2017). Cancer Susceptibility Gene Mutations in Individuals With Colorectal Cancer. Journal of Clinical Oncology, 35, 1086-1095.

Zhang X., et al. (1998). Structure of an XRCC1 BRCT domain: a new protein-protein interaction module. The EMBO Journal, 17(21), 6404–6411.

Zhang J., et al. (2015). Germline Mutations in Predisposition Genes in Pediatric Cancer.
N Engl J Med. 373(24):2336-2346.
